# BioVeL: a virtual laboratory for data analysis and modelling in biodiversity science and ecology

**DOI:** 10.1186/s12898-016-0103-y

**Published:** 2016-10-20

**Authors:** Alex R. Hardisty, Finn Bacall, Niall Beard, Maria-Paula Balcázar-Vargas, Bachir Balech, Zoltán Barcza, Sarah J. Bourlat, Renato De Giovanni, Yde de Jong, Francesca De Leo, Laura Dobor, Giacinto Donvito, Donal Fellows, Antonio Fernandez Guerra, Nuno Ferreira, Yuliya Fetyukova, Bruno Fosso, Jonathan Giddy, Carole Goble, Anton Güntsch, Robert Haines, Vera Hernández Ernst, Hannes Hettling, Dóra Hidy, Ferenc Horváth, Dóra Ittzés, Péter Ittzés, Andrew Jones, Renzo Kottmann, Robert Kulawik, Sonja Leidenberger, Päivi Lyytikäinen-Saarenmaa, Cherian Mathew, Norman Morrison, Aleksandra Nenadic, Abraham Nieva de la Hidalga, Matthias Obst, Gerard Oostermeijer, Elisabeth Paymal, Graziano Pesole, Salvatore Pinto, Axel Poigné, Francisco Quevedo Fernandez, Monica Santamaria, Hannu Saarenmaa, Gergely Sipos, Karl-Heinz Sylla, Marko Tähtinen, Saverio Vicario, Rutger Aldo Vos, Alan R. Williams, Pelin Yilmaz

**Affiliations:** 1 School of Computer Science and Informatics, Cardiff University, Queens Buildings, 5 The Parade, Cardiff, CF24 3AA UK; 2 School of Computer Science, University of Manchester, Kilburn Building, Oxford Road, Manchester, M13 9PL UK; 3 Institute for Biodiversity and Ecosystem Dynamics (IBED), University of Amsterdam, PO Box 94248, 1090 Amsterdam, The Netherlands; 4 Institute of Biomembranes and Bioenergetics (IBBE), National Research Council (CNR), via Amendola 165/A, 70126 Bari, Italy; 5 Department of Meteorology, Eötvös Loránd University, Pázmány sétány 1/A, Budapest, 1117 Hungary; 6 Department of Marine Sciences, University of Gothenburg, Box 463, 405 30 Gothenburg, Sweden; 7 Centro de Referência em Informação Ambiental, Avenida Dr. Romeu Tórtima, 388, Campinas, SP 13084-791 Brazil; 8 SIB Labs, Joensuu Science Park, University of Eastern Finland, P.O. Box 111, 80101 Joensuu, Finland; 9 Institute of Nuclear Physics (INFN), Via E. Orabona 4, 70125 Bari, Italy; 10 Max Planck Institute for Marine Microbiology, Celsiusstrasse 1, 28359 Bremen, Germany; 11 Jacobs University Bremen GmbH, Campus Ring 1, 28359 Bremen, Germany; 12 Stichting EGI (EGI.eu), Science Park 140, 1098 Amsterdam, The Netherlands; 13 Botanic Garden and Botanical Museum Berlin, Freie Universität Berlin, Königin-Luise-Strasse 6-8, 14195 Berlin, Germany; 14 IT Services, University of Manchester, Kilburn Building, Oxford Road, Manchester, M13 9PL UK; 15 Fraunhofer Institute for Intelligent Analysis and Information Systems (IAIS), Schloss Birlinghoven, 53757 Sankt Augustin, Germany; 16 Naturalis Biodiversity Center, Postbus 9517, 2300 Leiden, The Netherlands; 17 MTA-SZIE Plant Ecology Research Group, Szent István University, Páter K. u.1., Gödöllő, 2103 Hungary; 18 Institute of Ecology and Botany, Centre for Ecological Research, Hungarian Academy of Sciences, Alkotmány u. 2-4., Vácrátót, 2163 Hungary; 19 Swedish Species Information Centre/ArtDatabanken, Swedish University of Agricultural Sciences, Bäcklösavägen 10, 750 07 Uppsala, Sweden; 20 Department of Forest Sciences, University of Helsinki, P.O. Box 27, 00014 Helsinki, Finland; 21 Fondation pour la Recherche sur la Biodiversité (FRB), 195, rue Saint-Jacques, 75005 Paris, France; 22 Department of Biosciences, Biotechnology and Biopharmaceutics, University of Bari “A. Moro”, via Orabona, 1514, 70126 Bari, Italy; 23 Finnish Museum of Natural History, University of Helsinki, P.O. Box 17, 00014 Helsinki, Finland; 24 Institute of Biomedical Technology (ITB), National Research Council (CNR), via Amendola 122/D, 70126 Bari, Italy

**Keywords:** Biodiversity science, Ecology, Computing software, Informatics, Workflows, Virtual laboratory, Biodiversity virtual e-laboratory, Data processing, Analysis, Automation

## Abstract

**Background:**

Making forecasts about biodiversity and giving support to policy relies increasingly on large collections of data held electronically, and on substantial computational capability and capacity to analyse, model, simulate and predict using such data. However, the physically distributed nature of data resources and of expertise in advanced analytical tools creates many challenges for the modern scientist. Across the wider biological sciences, presenting such capabilities on the Internet (as “Web services”) and using scientific workflow systems to compose them for particular tasks is a practical way to carry out robust “in silico” science. However, use of this approach in biodiversity science and ecology has thus far been quite limited.

**Results:**

BioVeL is a virtual laboratory for data analysis and modelling in biodiversity science and ecology, freely accessible via the Internet. BioVeL includes functions for accessing and analysing data through curated Web services; for performing complex in silico analysis through exposure of R programs, workflows, and batch processing functions; for on-line collaboration through sharing of workflows and workflow runs; for experiment documentation through reproducibility and repeatability; and for computational support via seamless connections to supporting computing infrastructures. We developed and improved more than 60 Web services with significant potential in many different kinds of data analysis and modelling tasks. We composed reusable workflows using these Web services, also incorporating R programs. Deploying these tools into an easy-to-use and accessible ‘virtual laboratory’, free via the Internet, we applied the workflows in several diverse case studies. We opened the virtual laboratory for public use and through a programme of external engagement we actively encouraged scientists and third party application and tool developers to try out the services and contribute to the activity.

**Conclusions:**

Our work shows we can deliver an operational, scalable and flexible Internet-based virtual laboratory to meet new demands for data processing and analysis in biodiversity science and ecology. In particular, we have successfully integrated existing and popular tools and practices from different scientific disciplines to be used in biodiversity and ecological research.

**Electronic supplementary material:**

The online version of this article (doi:10.1186/s12898-016-0103-y) contains supplementary material, which is available to authorized users.

## Background

Environmental scientists, biologists and ecologists are pressed to provide convincing evidence of contemporary changes to biodiversity, to identify factors causing biodiversity decline, to predict the impact of, and suggest ways of combating biodiversity loss. Altered species distributions, the changing nature of ecosystems and increased risks of extinction, many of which arise from anthropogenic activities all have an impact in important areas of societal concern (human health and well-being, food security, ecosystem services, bioeconomy, etc.). Thus, scientists are asked to provide decision support for managing biodiversity and land-use at multiple scales, from genomes to species and ecosystems, to prevent or at least to mitigate such losses. Generating enough evidence and providing decision support increasingly relies on large collections of data held in digital formats, and the application of substantial computational capability and capacity to analyse, model, simulate and predict using such data [1–3]. Achieving the aims of the recently established Intergovernmental Science-Policy Platform on Biodiversity and Ecosystem Services (IPBES) [4] requires progressive developments in approach and method.

The complexity and scope of analyses in biodiversity science and ecology is growing very fast. It is becoming more common to carry out complex analysis using hundreds of data files with different structures and data types (e.g., genetic, species, geographical, environmental) combined with a variety of algorithms; producing results that need to be visualized in innovative ways. The requirement for scientists to work together, with collaborations that integrate datasets across many different parties and synthesize answers computationally to address larger scientific questions are becoming the norm. Biodiversity science and ecology are now in the era of data-intensive science [5, 6]. New research practices that productively exploit data pipelines and data-driven analytics need infrastructure that enables reliability, robustness, repeatability, provenance and reproducibility for large and complex scientific investigations. Methods evolve, exploiting tendencies to base on variants of previous processes, composed of common steps. However, usage statistics from developed science-wide e-Infrastructures show that biodiversity, conservation, and ecology scientists do not carry out large-scale experiments to the same extent as scientists in the physical sciences [7].

Scientific workflow systems, such as Kepler [8], Pegasus [9], Apache Taverna [10], VisTrails [11], KNIME [12], Galaxy [13] and RapidMiner [14] are mature technology for practical ways to carry out computer-based experimentation and analysis of relevant data in disciplines as diverse as medical ‘omics’/life sciences, heliophysics and toxicology [15–17]. Scientific workflow systems can be broadly organised into three categories. First, those developed for specialist domains, often with capabilities to be extended to other disciplines (e.g., LONI pipeline for neuro-imaging [18]; Galaxy for omics data processing; KNIME for pharmacological drug discovery). Secondly, there are workflow systems developed to be independent of any particular science discipline, with features for incorporating specialised customisations (e.g., Apache Taverna). Thirdly, there are those that cut across disciplines and focus on specific tasks (e.g., RapidMiner for data mining).

Workflows support both automation of routine tasks (data transformation, mining, integration, processing, modelling) and pursuit of novel, large-scale analysis across distributed data resources. Today, such activities are typically done in the R environment and here the integration with workflow systems can add value to current practice in ecological research. Most importantly, the record of what was done (the provenance) and the reproducibility of complex analyses can be enhanced when migrating ecological analysis into workflow environments, while workflow systems are able to handle the procedures for scaling computation on cloud infrastructure, for example. For this purpose, scientific workflow systems are starting to become used in biodiversity science and ecology for example: in ecology [19, 20], niche and distribution modelling [21–23], and digitisation of biological collections [24, 25].

Workflows can be deployed, executed, and shared in virtual laboratories. A modern virtual laboratory (sometimes also known as a virtual research environment, VRE) is a web-enabled software application that brings the digital, Internet-based data resources (which may include data collections, databases, sensors and/or other instruments) together with processing and analytical tools needed to carry out “in silico” or “e-science” work. As in a real laboratory, the essence of a virtual laboratory is providing the capability to carry out experimental work as a sequence of interconnected work processes i.e., a workflow. Data and tools are combined harmoniously to present a consistent joined-up computer-based work environment to the scientist user. The laboratory keeps track of the details of experiments designed and executed, as well as creating relevant provenance information about the data and tools used; to assist repeatability and replication of results. A virtual laboratory often also incorporates elements to provide assistance and to support collaborations between persons and across teams. These can include sharing and publishing mechanisms for data, experiments and results, as well as supplemental communications capabilities (either built-in or external) for Web-based audio/video conferencing, email and instant messaging, technical training and support.

The aim of our work has been to explore use of the workflow approach in ecology and to encourage wider adoption and use by developing, deploying and operating this Biodiversity Virtual e-Laboratory as a showcase for what is possible and as an operational service [26]. We have demonstrated this with results from a number of scientific studies that have used the BioVeL platform (Additional file 4).

## Implementation

The biodiversity virtual e-laboratory (BioVeL) provides a flexible general-purpose approach to processing and analysing biodiversity and ecological data (such as species distribution, taxonomic, ecological and genetic data). It is based on distributed computerised services accessible via the Internet that can be combined into sequences of steps (workflows) to perform different tasks.

**Fig. 1:**
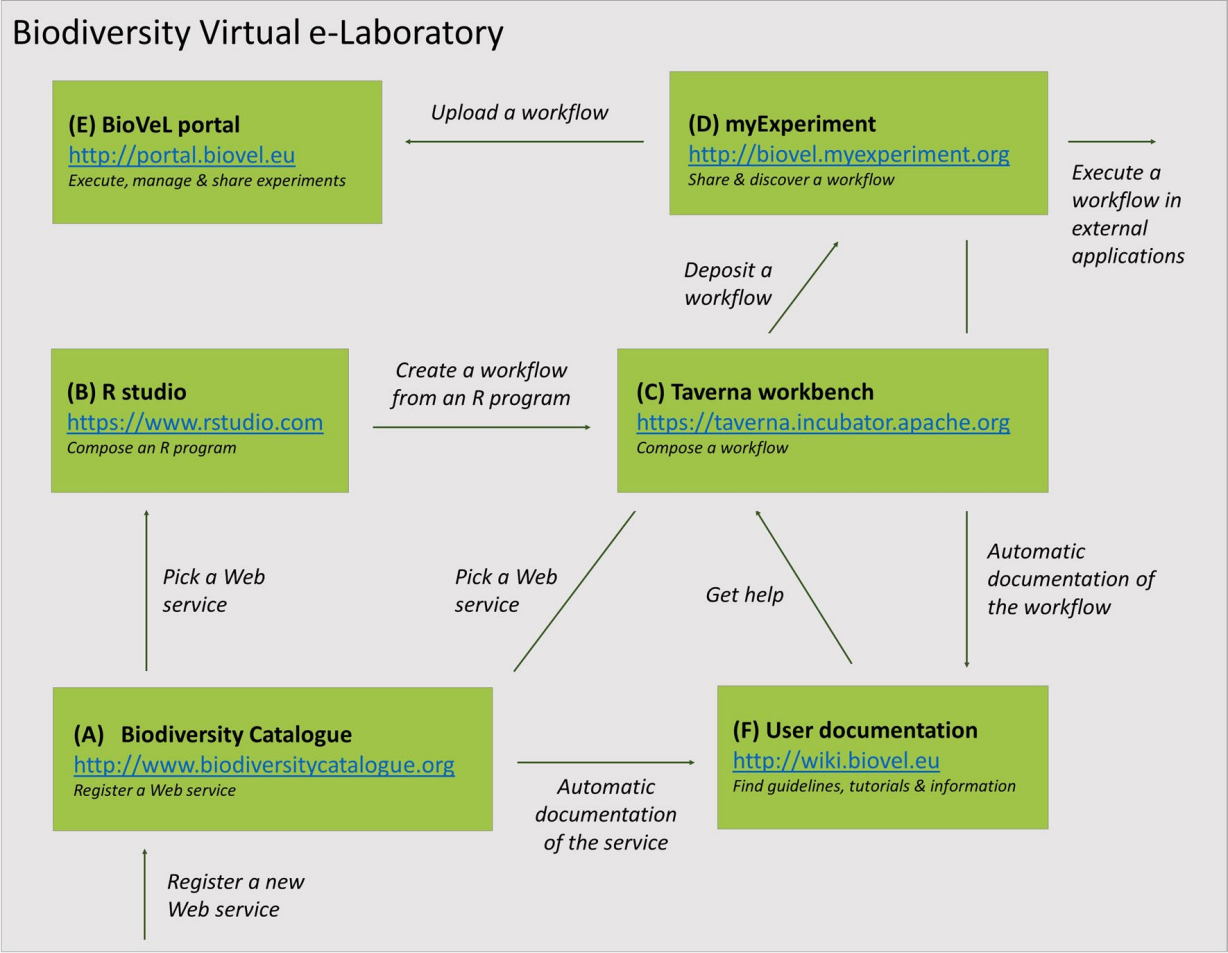
Biodiversity virtual laboratory (BioVeL) is a software environment that assists scientists in collecting, organising, and sharing data processing and analysis tasks in biodiversity and ecological research. The main components of the platform are: *A* the Biodiversity Catalogue (a library with well-annotated data and analysis services); *B* the environment, such as RStudio for creating R programs; *C* the workbench for assembling data access and analysis pipelines; *D* the myExperiment workflow library that stores existing workflows; *E* the BioVeL Portal that allows researchers and collaborators to execute and share workflows; and *F* the documentation wiki. Infrastructure is indicated in bold, while processes related to research activities are indicated in *italics*. Components *A*–*F* are referred to from the text, where they are described in detail. See also ‘how-to’ guidelines in the Additional information

The main components of the platform are illustrated in Fig. 1 and described following, with cross-references (A)–(F) between the text and the figure. Additional file 1 to the present article provides ‘how-to’ guidelines on how to make use of the various components.

### Web services for biodiversity science and ecology: (A) in Fig. 1

In computing terms, Web services are pieces of computing functionality (analytical software tools and data resources) deployed at different locations on the Internet (Worldwide Web) [27]. The idea of presenting data resources and analytical tools as Web services is an essential principle of the notion of the Worldwide Web as a platform for higher value “Software as a Service” applications, meaning users have to install less and less specialised software on their local desktop computers. Web services are central to the concept of workflow composition and execution; increasingly so with proliferation of third-party data resources and analytical tools, and trends towards open data and open science. Wrapping data resources and analytical tools to present the description of their interfaces and capabilities in a standard way aids the process of matching the outputs of one element in a workflow sequence to the inputs of the next. Where such matches are inexact, specialised services can be called upon to perform a translation function. Another benefit of describing resources and functions in a standardised way is the ability to register and advertise details in a catalogue akin to a ‘Yellow Pages’ directory, such that the resources and tools can be more easily discovered by software applications.

Many candidate Web services, representing useful biodiversity data resources and analytical tool capabilities can be identified from the different thematic sub-domains of biodiversity science. These include services coming from domains of enquiry such as: taxonomy, phylogenetics, metagenomics, ecological niche and population modelling, and ecosystem functioning and valuation; as well as more generally useful services relating to statistics, data retrieval and transformations, geospatial processing, and visualization. Working with domain experts via a series of workshops during 2012–2013 and other community networking mechanisms, we considered and prioritised more than 60 candidate services in seven groups (Table 1) many of which went on to be further developed, tested and deployed by their owning “Service Providers”. A full list of services is available in the Additional information.

**Table 1:** Services for data processing and analysis (Additional file 2)

Service group	Capabilities (web services)
General purpose, including mapping and visualization	General-purpose capabilities needed in many situations, such as for: Interactive visualization of spatio-temporal data (BioSTIF) e.g., occurrence data; Execution of R programs embedded as steps in workflows; Temporary workspace for data file movements between services
Ecological niche modelling	Built up from the existing openModeller web service [28] to offer a wide range of algorithms and modelling procedures integrated with geospatial management of environmental data, enabling researchers to create, test, and project ecological niche models (ENM)
Ecosystem modelling	A basic toolbox for studies of carbon sequestration and ecosystem function. It includes data-model integration and calibration services, model testing and Monte Carlo Experiment services, ecosystem valuation services, and bioclimatic services
Metagenomics	A basic set of services for studying community structure and function from metagenomic ecological datasets. It includes services for geo-referenced annotation, metadata services, taxonomic binning and classification services, metagenomic traits services, and services for multivariate analysis
Phylogenetics	Services to enable DNA sequence mining and alignment, core phylogenetic inference, tree visualization, and phylogenetic community structure, for broad use in evolutionary and ecological studies
Population modelling	Services for demographic data and their integration into matrix projection models and integral projection models (MPM, IPM)
Taxonomy	Services for taxonomic name resolution, checklists and classification, and species occurrence data retrieval

We have catalogued these capabilities (Web services) in a new, publicly available, curated electronic directory called the Biodiversity Catalogue (http://www.biodiversitycatalogue.org) [29]. This is an openly available online registry of Web services targeted towards the biodiversity science and ecology domain. It is an instance of software developed originally by the BioCatalogue project for the life sciences community [30], branded and configured for use in ecology. Our intention is that this catalogue should be well-founded through careful curation, and should become well-known and used, as is the case for the BioCatalogue in life sciences. The catalogue uses specialised service categories and tag vocabularies for describing services specific to biodiversity science and ecology and it has been operational since October 2012. Currently (date of writing), it catalogues 70+ services (some of which are aggregates of multiple capabilities) from 50+ service providers, including Global Biodiversity Information Facility (GBIF), European Bioinformatics Institute (EBI), EUBrazilOpenBio, PenSoft Publishers, Royal Botanic Gardens Kew, Species2000/ITIS Catalogue of Life (CoL), Pangaea, World Register of Marines Species (WoRMS), Naturalis Biodiversity Center and Canadensys). It has 130+ contributing members and is open for any provider of similar kinds of capabilities to register their Web services there.

The catalogue supports registration, discovery, curation and monitoring of Web services. Catalogue entries are contributed by the community and also curated by the community. Experts oversee the curation process to ensure that descriptions are high quality and that the services entries are properly annotated. We developed a 4-level service maturity model to measure the quality of service descriptions and annotations. Biodiversity Catalogue supports service ‘badging’ using this model. In this way, users can distinguish between services that are poorly described and perhaps unlikely to perform reliably, and those with higher quality descriptions and annotations. This encourages service providers to invest more time and effort in annotating their services and improving their documentation. It eases discovery and use of services by end users and scientists.

Within the catalogue we have provided an automated framework for service availability monitoring. Monitoring is performed on a daily basis. Service providers and curators are notified of potential availability problems when these are detected. The statistics collected over time are compiled into service reliability reports to give end users some indication of longer-term reliability of services and to help them choose the most reliable services for their scientific workflows and applications. This public portrayal of service performance information encourages Service Providers to invest time and effort in maintaining and improving the availability and reliability of their offering.

### Composing custom programs and workflows with Web services: (A), (B) and (C) in Fig. 1

Today, it is not only reliable and open Web services that are still scarce in ecology, but also easy-to-use applications and orchestration mechanisms that connect such services in a sequence of analytical steps. It takes significant effort to compose and prove an efficient workflow when the sequence of steps is complex—from tens to perhaps many hundreds of individual detailed steps. The inter-relations and transformations between components have to be properly understood to generate confidence in the output result.

In the R language [31] for example, interaction with servers via the HTTP protocol is built-in, so that a program client for a RESTful service only needs to compose the right request URL and decode the response. Both URL parameters and response formats are exhaustively documented in the Biodiversity Catalogue and off-the-shelf parsers exist for the syntax formats that our services return (e.g., JSON, CSV, domain-specific formats). For SOAP services, the R open source community uses the ‘S SOAP’ package to build more complex, stateful client–server interaction workflows, where an analysis is built up in multiple, small steps rather than a single request/response cycle.

The general-purpose Apache Taverna Workflow tool suite [10] is a widely used and popular approach to creating, managing and running workflows. With an established community of more than 7500 persons, organised into more than 300 specialised groups, having publicly shared more than 3700 workflows (information correct at March 2016) it represents a rich resource for scientists developing new analysis methods. We have chosen Apache Taverna as the basis for workflows that we developed to build on this already extensive platform, gaining advantage in expertise, familiarity, opportunities for cross-fertilisation and interdisciplinarity that increasingly characterises the science of biodiversity and ecology. With comprehensive capabilities to mix distributed Web Services, local programs/command line tools and other service types (e.g., BioMart queries or R programs) into a single workflow that can be executed locally, on specialised community or institutional computing facilities or “in the cloud”, Taverna was a suitable candidate for the task. We adapted the Taverna tools to meet new requirements we anticipated would arise during the course of this work.

We have developed appropriate interfaces in the Biodiversity Catalogue to invoke Web services directly from the R environment [32], or from within the Apache Taverna workflow management system [10]. We developed 20 interactive workflows to explore and showcase the utility of the Web services in ecological research. The workflows can be executed through the BioVeL Portal (E) described below. They are summarised in Table 2, with references to scientific studies using these workflows. A more detailed version of Table 2 is available as Additional file 3; also further Additional information describing different scientific studies that have made use of them.

**Table 2:** Workflows for biodiversity science (Additional file 3)

*Workflow (family)*	*Capability/purpose (i.e., what is it for?)* *incl. persistent identifier (purl) to locate the workflow and references to scientific studies that have exploited it*
Data refinement	The data refinement workflow (DRW) is for preparing taxonomically accurate species lists and observational data sets for use in scientific analyses such as: species distribution analysis, species richness and diversity studies, and analyses of community structure purl: http://www.purl.ox.ac.uk/workflow/myexp-2874.13 Portal: https://www.portal.biovel.eu/workflows/641 Scientific studies: [33, 34]
Ecological niche modelling (ENM)	The generic ENM workflow creates, tests, and projects ecological niche models (ENM), choosing from a wide range of algorithms, environmental layers and geographical masks purl: http://www.purl.ox.ac.uk/workflow/myexp-3355.20 Portal: https://www.portal.biovel.eu/workflows/440 The BioClim workflow retrieves environmentally unique points from a species occurrence file under a given set of environmental layers, and calculates the range of the environmental variables (min–max) for a given species purl: http://www.purl.ox.ac.uk/workflow/myexp-3725.2 Portal: https://www.portal.biovel.eu/workflows/443 Scientific studies: [33, 35, 36]
ENM statistical difference (ESW)	Statistical post-processing of results from ecological niche modelling ESW DIFF workflow computes extent, direction and intensity of change in species potential distribution through computation of the differences between two models, including change in the centre point of the distribution purl: http://www.purl.ox.ac.uk/workflow/myexp-3959.2 Portal: https://www.portal.biovel.eu/workflows/442 ESW STACK workflow computes extent, intensity, and accumulated potential species distribution by computing the average sum from multiple models purl: http://www.purl.ox.ac.uk/workflow/myexp-3856.3 Portal: https://www.portal.biovel.eu/workflows/70 Scientific studies: [33, 36]
Population modelling	Matrix population model construction and analysis workflows provide a complete environment for creating a stage-matrix with no density dependence, and then to perform several analyses on it. Each of the workflows in the collection is also available separately. The expanded version of this table, available as Additional information contains a link purl: http://www.purl.ox.ac.uk/researchobj/myexp-483 Portal: https://www.portal.biovel.eu/workflows/596 Integral projection models workflow provides an environment to create and test an integral projection model and to perform several analyses on that purl: http://www.purl.ox.ac.uk/researchobj/myexp-482 Portal: https://www.portal.biovel.eu/workflows/599 Scientific studies: no publication yet
Ecosystem modelling	Based around the Biome-BGC biogeochemical model, a collection of five workflows for calibrating and using Biome-BGC for modelling ecosystems and calculating a range of ecosystem service indicators. The Biome-BGC projects database and management system provides a user interface for setting of model parameters, for support sharing and reusing of datasets and parameter settings purl: http://www.purl.ox.ac.uk/researchobj/myexp-687 Portal: https://www.portal.biovel.eu/workflows/81 https://www.portal.biovel.eu/workflows/289 https://www.portal.biovel.eu/workflows/300 https://www.portal.biovel.eu/workflows/48 https://www.portal.biovel.eu/workflows/507 Scientific studies: [37–40]
Metagenomics	Microbial metagenomic trait calculation and statistical analysis (MMT) workflow calculates key ecological traits of bacterial communities as observed by high throughput metagenomic DNA sequencing. Typical use is in the analysis of environmental sequencing information from natural and disturbed habitats as a routine part of monitoring programs purl: http://www.purl.ox.ac.uk/workflow/myexp-4489.3 Portal: access on request (BioMaS) Bioinformatic analysis of Metagenomic ampliconS is a bioinformatic pipeline supporting biomolecular researchers to carry out taxonomic studies of environmental microbial communities by a completely automated workflow, comprehensive of all the fundamental steps, from raw sequence data arrangement to final taxonomic identification. This workflow is typically used in meta-barcoding high-throughput-sequencing experiments url: https://www.biodiversitycatalogue.org/services/71 Scientific studies: [41–43]
Phylogenetics	Bayesian phylogenetic inference workflows are for performing phylogenetic inference for systematics and diversity research. Bayesian methods guide selection of the evolutionary model and a post hoc validation of the inference is also made. Phylogenetic partitioning of the diversity across samples allows study of mutual information between phylogeny and environmental variables purl: http://www.purl.ox.ac.uk/researchobj/myexp-370 Portal: https://www.portal.biovel.eu/workflows/466 https://www.portal.biovel.eu/workflows/549 https://www.portal.biovel.eu/workflows/550 https://www.portal.biovel.eu/workflows/525 PDP workflow, using PhyloH for partitioning environmental sequencing data using both categorical and phylogenetic information purl: http://www.purl.ox.ac.uk/workflow/myexp-3570.5 Portal: https://www.portal.biovel.eu/workflows/434 https://www.portal.biovel.eu/workflows/71 MSA-PAD workflow performs a multiple DNA sequence alignment coding for multiple/single protein domains invoking two alignment modes: gene and genome Gene mode purl: http://www.purl.ox.ac.uk/workflow/myexp-4549.1 Portal: https://www.portal.biovel.eu/workflows/712 (access on request) Genome mode purl: http://www.purl.ox.ac.uk/workflow/myexp-4551.1 Portal: https://www.portal.biovel.eu/workflows/713 (access on request) SUPERSMART (self-updating platform for estimating rates of speciation and migration, ages and relationships of taxa) is a pipeline analytical environment for large-scale phylogenetic data mining, taxonomic name resolution, tree inference and fossil-based tree calibration url: https://www.biodiversitycatalogue.org/services/78 Scientific studies: [44–47]

### Creating R programs that use Web services: (B) in Fig. 1

Users can interact with the Biodiversity Catalogue and its services in a variety of ways, one of which is by developing their own analysis programs that invoke services in the catalogue. Both the catalogue itself and the services that are advertised in it are exposed through Applications Programming Interfaces (API) that are accessible using standard Internet protocols (HTTP, with RESTful or SOAP functionality). Hence, writing custom analysis code is relatively straightforward in commonly-used programming languages, such as R [31]. The advantage of this way of interacting with the Biodiversity Catalogue services is that users can do this within a development environment (such as RStudio [48]). This enables them to go through their analysis one step at a time (in a “read-eval-print loop”) visually probing their data as it accumulates. Users can include additional functionalities accessible through Web services [49] as well as from relevant third-party R packages for biodiversity and ecological analysis; many of which have been developed in recent years. These latter are available, for example via CRAN [50]. The Additional file 1 ‘how-to’ guidelines points to an example of how to create an R program that calls a Web service. Given the popularity of the R programming language in biodiversity and ecology, we expect to see not just ad hoc analysis programs but also published, re-usable analysis libraries written against Web services APIs. The Biodiversity Catalogue provides a single place where such Web services can be found.

Several Web services can be linked together in sequence in an R program to create a ‘work flow’. However, this can rapidly become quite complex. Outputs of one Web service may not match the inputs of the next. Conversions and other needs (such as conditional branching, nesting of sub-flows, parallel execution of multiple similar steps, or waiting asynchronously for a long-running step to complete) all add to the complexity, which has to be managed. Here, workflow management systems, like Apache Taverna [10] can hide some of the complexity and make workflows easier to create, test and manage. Such systems often offer graphical ‘what you see is what you get’ user interfaces to compose workflows from Web and other kinds of services, such as embedded R programs. Reasonably complex custom workflows can be created (see below) without writing a single line of programming code, which can be attractive for scientists with little or no programming background.

Combined with other capabilities of the BioVeL platform, including transparent access to greater levels of computing capability and capacity for processing large amounts of data, managing the complexity of multiple workflow runs, sharing workflows and provenance, offering data services, etc.) Web services can be applied (i) consistently and (ii) in combination. We have given further examples of potential areas of application where these functions can be combined to support and accelerate new research in the "Discussion" section below (under ‘Towards more comprehensive and global investigations’).

### Creating a workflow from an R program: (B) and (C) in Fig. 1

It is possible to convert pre-existing R programs for inclusion into Taverna workflows as discrete ‘R service’ steps. We have developed some recommendations [51] to make this as easy as possible. We have, for example taken an existing R program that uses data from a local directory and incorporated this into a workflow that generates graphical plots from Ocean Sampling Day (OSD) data [41] to visualise the metagenomic sequence diversity in ocean water samples. We exposed the inputs and outputs of the R program as ‘ports’ of the corresponding R service, such that the program can be easily re-run using different data. A user could re-use this or another R program, wrapped as a service into their own workflow. Because the workflow is executed on the BioVeL platform, including execution of the R service, there is no need to run R locally on their own computer. This approach gives the possibility to combine R programs and workflows in complementary fashion, the full power of which becomes evident when workflows are embedded as executable objects in 3rd party web sites and web applications (see "Execute a workflow in external applications" section, below).

### Building a workflow from Web services: (C) in Fig. 1

Using the Apache Taverna Workbench [10], we devised workflows meeting scientists’ own needs or fulfilling common needs for routine tasks performed by many scientists in a community. The design and creation of a workflow from Web services requires some programming skills and has often been done by service curators at institutes that also provide Web services.

The Taverna Workbench is a ‘what you see is what you get’ graphical tool, locally installed on the user’s desktop computer that can be used to create and test workflows using a ‘drag and drop’ approach. In the Workbench, users select processing steps from a wide-ranging list of built-in local processing steps and on-line Web services to create a workflow. They do this by dragging and dropping the step into a workflow and linking to its other steps. Each step is in reality an encapsulation of a software tool (an R program, for example) with its own inputs and outputs. Workbench users link the inputs of a step to the outputs from a preceding step and the outputs to the inputs of the next step. Links can be edited when steps are inserted, removed or re-organised. The user can test the workflow by running it locally on their desktop computer or by uploading it to BioVeL Portal (described below).

We have provided a customised version of the Workbench, Taverna Workbench for Biodiversity [52], configured with a selectable palette of services especially relevant to biodiversity science and ecology. This version provides a direct link to the Biodiversity Catalogue, allowing users to search for the most recent and useful external Web services provided by the community as a whole.

### Customising existing workflows: (D) and (C) in Fig. 1

Scientists with programming skills can inspect and modify existing workflows available in the myExperiment workflow library (http://biovel.myexperiment.org), again using the Taverna Workbench tool. There is a direct link to the public myExperiment workflow library (described below), allowing to search for and download existing workflows.

As an example, a scientist used an existing workflow for statistical calculations of differences between ENM output raster files (Table 2, ESW DIFF) to create a new variant that additionally calculates the magnitude and direction of shift in distribution between two model projections. Enhancing the underlying logic (R program in this case) with additional code to compute the weighted centre point of each model projection, and the geographic distance between them was all that was needed. The required data management and visualization resources were already in place, provided by other elements of the existing workflow and the BioSTIF service (Table 1). The ESW DIFF workflow was modified to include the functionality of the new variant.

In a further example: Aphia, the database underlying the World Register of Marine Species (WoRMS) [53] is a consolidated database for marine species information, containing valid species names, synonyms and vernacular names, higher taxon classification information and extra information such as literature and biogeographic data. Its principal aim is to aid data management, rather than to suggest any taxonomic or phylogenetic opinion on species relationships. As such it represents a resource that is complementary to those already programmed as part of the Data Refinement Workflow (Table 2). After working with the Service Provider to register the service in the Biodiversity Catalogue, we easily modified the Data Refinement Workflow to present the AphiaName lookup service as a choice alongside the Catalogue of Life and GBIF Checklist Bank lookup services when carrying out the taxonomic name resolution stage of the workflow.

### Building and using workflow components (not illustrated in Fig. 1)

Packaging a series of related processing steps into a reusable component eases the complexity of building workflows. For example, the task of dynamically defining a geographically bounded area (known as a mask) within which something of interest should be modelled (or selecting from a list of pre-defined masks) involves a lengthy sequence of steps and interactions between a user and a Web service that is used to do the mask creation and selection. To create this from scratch every time it is needed in a workflow would be time-consuming and error-prone. A “create_or_select_mask” component makes it easier to do.

Such components serve as basic building blocks in larger or more complex workflows, making workflows quicker and easier to assemble. We have developed a series of ecological niche modelling (ENM) components that have been mixed and matched for investigating the effects of mixing different spatial resolutions in ENM experiments [35]; as well as to assemble a jack-knife resampling workflow to study the influence of individual environmental parameters as part of our study on species distribution responses to scenarios of predicted climate change [54]. The packages of population modelling workflows (Table 2) are also based on component families, allowing mix-and-match configuration of population modelling analyses. Well-designed and well-documented sets of workflow components can effectively allow a larger number of scientists without in-depth programming skills to more easily assemble new analytical pipelines.

### Discovering workflows: (D) in Fig. 1

As with making Web services available in a directory to encourage discovery and re-use, sharing workflows publicly encourages re-use and adoption of new methods. It makes those methods available to users having less skills or time and effort to create such methods. More importantly, sharing enables more open science, repeatability and reproducibility of science, as well as favouring peer-review of both the methods themselves and results arising from their use.

One mechanism for sharing nurtures a distinctive community of biodiversity workflow practitioners within the well-established myExperiment online workflows repository [55]. This social repository provides workflow publishing, sharing and searching facilities. Within myExperiment we have established a discrete group with its own distinctive branding, where our workflows are shared. The BioVeL group [56] allows scientists from the biodiversity community to upload their workflows, in silico experiments, results and other published materials. Currently (at the time of writing) the BioVeL domain of myExperiment features almost 40 workflows. Through active participation and collaboration, users can contribute to and benefit from a pool of scientific methods in the biodiversity domain, and be acknowledged when their workflows have been re-used or adapted.

### Executing workflows: (E) in Fig. 1

As a part of the BioVeL virtual laboratory, we designed and deployed the BioVeL Portal (http://portal.biovel.eu) [26], an Internet Web browser based execution environment for workflows. The Portal does not require any local software installations and scientists can use a Web browser interface to upload and execute workflows from myExperiment (or they can choose one already uploaded). Once initiated, users are able to follow the progress of the analysis and interact with it to adjust parameters or to view intermediate results. When satisfied with the final results, a user can share these with others or download them to their local computer. Results can be used as inputs to subsequent work or incorporated into publications, with citation to the workflow and parameters that produced them.

We adopted and adapted SEEK, the systems biology and data model platform [57] to meet the needs of the biodiversity science and ecology community. We re-branded SEEK for BioVeL and gave it a user interface suited to typical tasks associated with uploading and executing workflows and managing the results of workflow runs. We equipped it to execute workflows on the users’ behalf, for multiple users and multiple workflows simultaneously.

The BioVeL Portal offers functions for discovering, organising and sharing both blueprints for analyses (i.e., workflows) as well as results of analyses (i.e., workflow runs) among collaborators and groups. The Portal provides users with their own personal workspace in which to execute workflows using their own data and to keep their results. Users can manage how their results are shared. At any time, they can share workflows and results publicly, within and between projects, or in groups of individuals. Users can return to their work at any time and pick up where they left off. This ability to create ‘pop-up’ collaborations by inviting individuals into a shared workspace to explore an emerging topic, and to keep track of work offers an immediate way to establish exciting new collaborations with little administrative overhead.

Presently (at the time of writing) there are 50 workflows publicly available within the BioVeL Portal. To support them we have provided a Support Centre, including training materials, documentation (http://wiki.biovel.eu) and helpdesk (mailto:support@biovel.eu) where users can obtain assistance. The expanded version of Table 2 in the Additional file 3 gives full details.

As an example, workflows created for invasive alien species studies [54] have been frequently re-used in other scientific analyses; for example, to predict potential range shifts of commercially important species under scenarios of climatic change [36], and to describe the biogeographic range of Asian horseshoe crabs [58]. Here especially, seamless linkage of data access to species occurrence records and environmental data layers, as well as the partly automated cleaning and processing procedures are useful functions when running niche modelling experiments for several species across a large number of parameter settings. The Data Refinement Workflow [59] has likewise been used in both preparation of niche modelling experiments as well as in analysis of historical changes in benthic community structure [34]. Here especially, the Taxonomic Name Service and data cleaning functions were helpful in resolving synonyms, correcting misspellings, and dealing with other inconsistencies in datasets compiled from different sources.

The Portal also offers functions for data and parameter sweeping. This includes batch processing of large quantities of separate input data using the same parameters (data sweeping) and batch processing the same data using different parameters (parameter sweeping). As example, the niche modelling workflow (Table 2, ENM) has 15 user interaction steps where parameters or files have to be supplied. When repeated manually multiple times this is error-prone. The sweep functions can be used to automate systematic exploration of how data and parameters affect the results in a larger analysis. In such cases the Portal can automatically initiate multiple workflow runs in parallel, significantly reducing the time needed to complete all the planned experiments. It is possible to delegate computing intensive operations to 3rd party computing facilities such as a high performance computing (HPC) centre or a cloud computing service.

Scientists have used the batch processing capability of BioVeL to explore parameter space in models and to generate comparable results for a large number of species. For example, in investigations of present and future distributions of shellfish (Asian Horseshoe Crabs) under predicted climate changes, the technique has been used to generate consensus outputs based on several different, individually executed niche modelling algorithms (for example: MaxEnt, Support Vector Machine and Environmental Distance) to build and evaluate a wide range of models with different combinations of environmental data layers (parameter sweep with 12 different combinations of environmental layers); and to build models for multiple ecologically similar species (data sweep for six intertidal shellfish species). Such calculations, running three modelling algorithms with 12 different environmental datasets for six species (i.e., 216 models) can be concluded in a single day via the Portal.

### Execute a workflow in external applications

Finally, BioVeL supports executable workflows to be embedded in other web sites and applications; just like YouTube™ videos can be embedded in web sites. Such embedding would allow, for example a web site giving statistical information about fluctuations in a species population to be rapidly updated as soon as the most recent survey data is entered. Or, it could allow members of the public (e.g., school students) to explore ‘what-if’ scenarios by varying the data and parameter detail without specific knowledge of the workflow executing behind the website. In Scratchpads [60], 6000+ users have the possibility now to embed workflows into their personal and collaborative Scratchpad websites to repeatedly process their data; as in BioAcoustica [61] for example. BioAcoustica is an online repository and analysis platform for scientific recordings of wildlife sounds. It embeds a workflow based on an R package that allows scientists contributing data to the site to analyse the sounds.

### Distributed computing infrastructure and high performance computing

Although not reported in detail in the present paper, we configured and deployed the underlying information and communications technology (ICT) infrastructure needed to support a multi-party distributed heterogeneous network of biodiversity and ecology Web services (the Biodiversity Service Network), and the execution of workflows simultaneously by multiple users. We offered a pilot operational service. In doing so we utilised different kinds of distributed computing infrastructure, including: Amazon web services (AWS), EGI.eu Federated Cloud/INFN ReCaS Network Computing, SZTAKI Desktop Grid, as well as various localised computer servers under the administration of the partner and contributing organisations. This demonstrates the ability of the BioVeL Web services Network to cope with heterogeneity of underlying infrastructures by adopting a service-oriented computing approach.

## Discussion

### Principal findings

We wanted to kick-start familiarisation and application of the workflow approach in biodiversity science and ecology. Our work shows that the Biodiversity Virtual e-Laboratory (BioVeL) is a viable operational and flexible general-purpose approach to collaboratively processing and analysing biodiversity and ecological data. It integrates existing and popular tools and practices from different scientific disciplines to be used in biodiversity and ecological research. This includes functions for: accessing data through curated Web services; performing complex in silico analysis through exposure of R programs, workflows, and batch processing functions; on-line collaboration through sharing of workflows and workflow runs; experiment documentation through reproducibility and repeatability; and computational support via seamless connections to supporting computing infrastructures. Most of these functions do exist today individually and are frequently used by biodiversity scientists and ecologists. However, our platform unites them as key components of large-scale biodiversity and ecological research in a single *virtual research environment*.

We developed scientifically useful workflows in thematic sub-domains (taxonomy, phylogenetics, metagenomics, niche and population modelling, biogeochemical modelling) useful to address topical questions related to ecosystem functioning and valuation, biospheric carbon sequestration and invasive species management. These topical science areas have real unanswered scientific questions, with a potentially high societal impact arising from new knowledge generated. We applied our workflows to case studies in two of these areas, as well as to case studies more generally in niche modelling and phylogenetics. Our scientific results (Additional file 4) demonstrate that the combination of functions in BioVeL have potential to support biodiversity and ecological research involving large amounts of data and distributed data, tools and researchers in the future.

### Strengths and weaknesses

#### Productivity gains

The key criterion for success of the infrastructure and the associated use of Web services is delivering the ability to perform biodiversity and ecology research faster, and/or cheaper, and/or with a higher quality. From the scientists’ perspective, we have seen increased ease of use and improved ability to manage complexity when faced with manipulation and analysis of large amounts of data. The upfront investment to design new workflows pays off not only in the multiple applications of it to different scientific questions and re-uses of it across data and parameter sweeps; but also in terms of time to accomplish work, especially when large analysis can be easily delegated to appropriate computing infrastructures.

Exploiting distributed data resources and processing tools via the Internet opens access to vastly greater computing capacity and analytical capability than is normally available in a desktop or local cluster computer. Our work with the Biome-BGC workflows (see Additional file 4) model and supporting database reused 1100 datasets and 84 parameter sets 84 times, achieving a performance of about 92,000 model runs during 22 days (three simulations per minute on average).

#### Meeting conditions for reproducibility of work

Wrapping R programs and Web service interactions in workflows removes the repetitiveness, inconsistency and lack of traceability of manual work, while permitting consistent repetition of an experiment. The BioVeL system keeps track of how the analysis was done, documents the research steps and retains the provenance of how the workflow executed. This provenance information helps in recording and tracing back to decisions, reducing time for error discovery and remedy; as well as formalization for reporting. It is these consistent processing and tracking features (rather than speed of execution per se) that are a principal advantage when dealing with large amounts of data, and when running many algorithms and different parameter settings across that data. They give an investigator the ability to document, overview, share and collaboratively evaluate the results from a complex large-scale study.

A progressive drive towards more open research, including with greater reproducibility [62, 63] and stronger emphasis on ‘*elevating the status of code in ecology*’ [64] is leading journal publishers (including those of the present article, BioMed Central) to make it a condition of publication that data (and increasingly, software) should be accessible and easy to scrutinise. As noted in a BMC Ecology editorial [65] the idea that the data underlying a study should be available for validation of conclusions is not unreasonable. By implication, “*…readily reproducible materials… freely available…*” includes the workflows and software that have been used for preparation and analysis of that data. Using the BioVeL ecosystem is an easy way of meeting such conditions.

#### Increased levels of inter-disciplinary working

The infrastructure enables increased levels of inter-disciplinary working and more scalable scientific investigations. The first generation of publications resulting from the e-laboratory is encouraging and shows that BioVeL services start providing these features. The majority of the users of ecological niche modelling workflows (for example) may not be experts in this field. They can be scientists with backgrounds in ecology, systematics, and environmental sciences that use the workflows to become familiar with new analytical methods [33, 36, 54]. Similarly, the taxonomic, phylogenetic and metagenomic services have been used by scientists to complement their existing analytical expertise with that from another field [36, 44]. A further example: Amplicon-based metagenomics approaches have been widely used to investigate both environmental and host associated microbial communities. The BioMaS (Bioinformatic analysis of Metagenomic ampliconS) Web service (Table 2; [43]) offers a way to simply and accurately characterize fungal and prokaryotic communities, overcoming the necessity of computer-science skills to set up bioinformatics workflows. This is opening the field to a wide range of researchers, such as molecular biologists [66] and ecologists [42].

#### Towards more comprehensive and global investigations

The principal BioVeL functionalities support more comprehensive and global investigations of biodiversity patterns and ecological processes. Such investigations are not impossible today but they are expensive and often can only be addressed with large and resourceful scientific networks. Exploiting such scalability is particularly attractive, for example to prepare and verify large-scale data products relating to the essential biodiversity variables (EBV) [67]; for phyloclimatic investigations [68]; and for characterisation of biogeographic regions [69]. In addition, complex predictive approaches that couple mechanistic with statistical models may benefit from the use of the BioVeL environment [70]. All these kinds of processing usually require integration of distributed biological, climate and environmental data, drawn from public databases as well as personal sources. They depend on a wide range of analytical capabilities, computational power and, most importantly the combined knowledge of a large number of experts. The BioVeL platform can connect these critical resources on the fly. In conjunction with an easy-to-use interface (the Portal) they can be used to dynamically create ad hoc scientific networks and cross-disciplinary collaborations fast. In the absence of dedicated funding it is a mechanism that can help scientists to react more quickly to newly emerging socio-environmental problems. The infrastructure is increasingly used for this purpose of ‘next-generation action ecology’ [71].

#### Dependency on supporting infrastructure and robust Web services

One apparent drawback of the approach we describe is dependency on the ready availability of robust infrastructure to provide access to data and to processing capabilities. This is out of the control of the end-user scientists but it is a matter for service providers. It is the same issue we face as consumer users of the Internet, whereby we rely on a well-developed portfolio of robust related services; for example, for making our travel arrangements with airlines, rental cars and hotels. In the biodiversity and ecology domain this is not the case. The portfolio of services is not yet well developed. There are only a limited number of robust large-scale service providers thus far (GBIF, EMBL-EBI, OBIS, PANGAEA to name just four examples) and not many smaller ones. Compare this with the life sciences community, where more than 1000 Web services from more than 250 service providers are listed in the BioCatalogue [30]. By promoting the Biodiversity Catalogue [29] as the well-founded one-stop shop to keep track of high-quality Web services as they appear; and annotating entries in the catalogue to document their capabilities we are hoping to encourage steps towards greater maturity. As with all software, the services and workflows, and the platforms on which they run have to be maintained. There is a cost associated with that. Projects like Wf4ever (“Workflow for Ever”) [72] have examined some of the challenges associated with preserving scientific experiments in data-intensive science but long-term it is a community responsibility that still has to be addressed.

## Results in context

### Prototypes to operational service

Historical projects such the UK’s BBSRC-funded Biodiversity World project, 2003–2006 [73] and the USA’s NSF-funded SEEK project 2002–2007 [74] (not to be confused with the SEEK platform for systems biology) successfully explored the potential of automated workflow systems for large-scale biodiversity studies. Moving from concept-proving studies towards a reliable infrastructure supporting collaboration is a substantial challenge. In the long-run such infrastructure has to robustly serve many thousands of users simultaneously.

With BioVeL we offer a pilot-scale operational service, delivered continuously and collaboratively by multiple partner organisations. This “Biodiversity Commons” of workflows, services and technology products can be used by anyone. Embedding elements of it within third party applications and contexts such as Scratchpads [75], Jupyter/iPython Notebooks [76], data analysis for Ocean Sampling Day collection events [41], national level biodiversity information infrastructures [77] and biodiversity observation networks has a multiplier effect, making it possible for all users of those wider communities and others to execute and exploit the power of workflows.

The underlying SEEK platform [57] on which BioVeL is based (not to be confused with the SEEK project mentioned above) is designed fundamentally to assist scientists to organise their digital data analysis work. As well as supporting execution of workflows, it allows them to describe, manage and execute their projects. These normally consist of experiments, datasets, models, and results. It helps scientists by gathering and organising pieces of information related to these different artefacts into different categories and making links between them; namely: yellow pages (programmes, people, projects, institutions); experiments (investigations, studies, assays); assets (datasets, models, standard operating procedures, publications); and activities (presentations, events). Not all the functionality of SEEK is presently enabled in the BioVeL Portal variant but in future it can be enabled as the needs of the community grow.

### Global research infrastructures

Globally, organisations with data and processing facilities across the world are working to deliver research infrastructure services to their respective scientific user communities. Initiatives in Europe (LifeWatch), Australia (Atlas of Living Australia), Brazil (speciesLink network, SiBBr Brazilian Biodiversity Information System), China (Academy of Sciences National Specimen Information Infrastructure and the World Federation of Culture Collections), South Africa (SANBI Integrated Biodiversity Information System), USA (DataONE and NEON) as well as GBIF, Catalogue of Life, Encyclopedia of Life, Biodiversity Heritage Library, and others are all mutually interdependent. They are driven not only by the direct needs of curiosity science but also more and more by the science needs of global policy initiatives. All research infrastructure operators recognise the need to remove barriers to global interoperability through common approaches based on interoperable Web services and promoting the development, sharing and use of workflows [78]. Our work is relevant to and supports this goal.

### IPBES, GEO BON, and essential biodiversity variables

The Intergovernmental Science-Policy Platform on Biodiversity and Ecosystem Services (IPBES) has to provide assessments of the state of the environment [4]. Guidelines for authors of assessments focus on several areas highly relevant in the context of the present paper: (i) improving access to data, information and knowledge of all types; (ii) managing data uncertainty and quality; and (iii) performing various model simulations and scenario-based analysis of future developments [79]. Additionally, some key principles and practices are given to ensure respect for and to consistently apply transparency at all steps of data collection, selection, analysis and archiving. This is so that IPBES can enable replication of results and informed feedback on assessments; comparability across scales and time; and use of systematic methodology and shared approach in all steps of the assessment process. The workflow approach, applied via BioVeL tools and infrastructure with specific additional developments to support Essential Biodiversity Variables in conjunction with other partners from the Group on Earth Observations Biodiversity Observation Network (GEO BON) would be a very progressive move to fulfil these requirements [80].

### Towards wider use of workflows

Tools for creating, executing and sharing workflows to process and analyse scientific data (see third paragraph of the introduction) have been around for 15 years. Most of these started life as desktop tools. Indeed, Kepler was a product of the previously mentioned SEEK project [74], with origins in ecological science. Despite variable usage across disciplines the cumulative experience is that the general approach of configurable, flexible workflows to assist the process of transforming, analysing and modelling with large amounts of data is well accepted. Workflows as a paradigm for orchestrating disparate capabilities to pursue large-scale data intensive ecological science are an important next step for the community. They represent “*primacy of method*” for a community evolving towards a new research culture that is increasingly dependent on working collaboratively, exchanging and aggregating data and automating analyses [63, 81]. They balance shareability, repeatability and flexibility with simplicity.

## Conclusions

In conclusion, we have presented a virtual laboratory that unites critical functions necessary for supporting complex and data intensive biodiversity science and ecological research in the future. We have created and deployed multiple Web services and ‘off-the-shelf’ packs of pre-defined workflows that meet the specific needs for several types of scientific study in biodiversity science and ecology. We have made these available respectively through a catalogue of services, the Biodiversity Catalogue and via a public repository of workflows, myExperiment. Each part can be used independently of the others or as an integrated part of the platform as a whole. BioVeL is operational and we have provided guidelines for its use (Additional file 1). We can refer (via Additional file 4) to many scientific studies that have used and are using the platform. We have raised awareness of what is possible and have laid foundations for further adoption and convergence activities as more ecologists encounter the worlds of big data and open science.

### We foresee two main directions of future development

Firstly, building complete, flexible, independent virtual laboratories will become more commonplace. Scientists want to be in control of their own real physical laboratories and there is no reason to assume they will not want to be in control of their own virtual laboratories for data processing and analysis. As with their physical laboratories, scientists will not want to build all elements from scratch. They will wish to take advantage of proven ready-built workflows and workflow components built and tested by trusted suppliers. Such workflows and components are part of an emerging Biodiversity Commons those labs can draw upon. We already have the first cases where scientists use BioVeL to expose and share their own analytical assets, and begin to pool and aggregate tools developed by the community rather than for the community. Capabilities for data management have been built into the core of BioVeL because the users requested it. Combined, such functionality provides a comprehensive collaborative platform that supports the needs of modern-day reproducible digital science.

The second direction extends towards building the sufficient base of robust data and computational services in biodiversity and ecological sciences (Web services, R programs and command line tools) that can be combined to automate multi-stage processing and analysis tasks. This will give scientists the freedom to compose sequences of processing steps to perform the scientific tasks they know and are familiar with today. At the same time the approach retains a dual flexibility. It permits the addition of new capabilities as those develop and evolve. It also allows for composing capabilities in ways that cannot presently be foreseen; thus meeting scientists’ needs of the future.

## Additional files

**Additional file 1.** ‘How-to’ guidelines for the biodiversity virtual e-laboratory.

**Additional file 2: Table S1.** Service groups and capabilities for processing and analysis in biodiversity science.

**Additional file 3: Table S2.** Workflows for biodiversity science.

**Additional file 4.** Scientific studies using the BioVeL platform.

## Abbreviations

API: application programming interface
BioMaS: bioinformatic analysis of metagenomic ampliconS
BioVeL: biodiversity virtual e-laboratory
BPI: Bayesian phylogenetic inference
CoL: Species 2000/ITIS catalogue of life
CRAN: comprehensive R archive network
CRIA: Centro de Referência em Informação Ambiental
DataONE: data observation network for earth
DRW: data refinement workflow
EBI: European Bioinformatics Institute
EBV: essential biodiversity variable
EMBL: European Molecular Biology Laboratory
EMBL-EBI: EMBL European Bioinformatics Institute
ENM: ecological niche modelling
ESI: ecosystem service indicator
ESW: ENM statistical difference workflow
GBIF: Global Biodiversity Information Facility
GEO BON: Group on Earth Observations Biodiversity Observation Network
HPC: high performance computing
IPBES: Intergovernmental Science-Policy Platform on Biodiversity and Ecosystem Services
IPM: integral projection model
LONI: Laboratory of Neuro Imaging
MMT: microbial metagenomic trait calculation and statistical analysis workflow
MPM: matrix projection model
MSA-PAD: multiple sequence alignment based on PFAM accessed domain information
NEON: National Ecological Observatory Network
OBIS: Ocean Biogeographic Information System
OSD: Ocean Sampling Day
OTU: operational taxonomic unit
PURL: persistent uniform resource locators
REST: representational state transfer (a mechanism for communicating between application programs and some web services cf. SOAP)
SANBI: South African National Biodiversity Institute
SEEK: science environment for ecological knowledge (historical project)
SEEK: systems biology and data model platform (unrelated to SEEK, the project)
SOAP: simple object access protocol (a mechanism for communicating between application programs and some web services cf. REST)
VRE: virtual research environment
WoRMS: World Register of Marine Species
