# Supplementary material for: BioVeL: a virtual laboratory for data analysis and modelling in biodiversity science and ecology
Source: BMC Ecol. 2016 Oct 20;16:49. doi: 10.1186/s12898-016-0103-y (PMC5073428; doi:10.1186/s12898-016-0103-y)
Supplement: Supplementary file 1 — Additional file 1. ‘How-to’ guidelines for the biodiversity virtual e-laboratory. [file 12898_2016_103_MOESM1_ESM.docx]

**Supplementary Information**

**‘How-to’ guidelines for the Biodiversity Virtual e-Laboratory**

**Getting started**

*The BioVeL wiki (*[*https://wiki.biovel.eu/*](https://wiki.biovel.eu/)*) contains lots of help on how to use BioVeL including the following key resources to help you get started:*

- *Working with BioVeL*

[*https://wiki.biovel.eu/display/doc/Working+with+BioVeL*](https://wiki.biovel.eu/display/doc/Working+with+BioVeL)

- *Training manual – Ecological Niche Modelling and related workflows, which will introduce you to most aspects of running workflows in the BioVeL portal*

[*https://wiki.biovel.eu/display/doc/Training+manual+-+Ecological+Niche+Modelling+and+related+workflows*](https://wiki.biovel.eu/display/doc/Training+manual+-+Ecological+Niche+Modelling+and+related+workflows)

*All workflows come with their own demonstration / tutorial, including a default set of data and starting parameter settings so that you can run any workflow as an example, without having to supply your own data. These tutorials can be found in the wiki pages for the particular workflow of interest.*

*Further pointers to specific help information relating to the main components of the platform illustrated in Figure 1 are listed below.*

*If you cannot find information you think should be in the Wiki, or something is not sufficiently clearly explained, please contact us by email to* [*support@biovel.eu*](mailto:support@biovel.eu)

*You may also be interested in:*

- *GBIF Guide to Data Refinement Using the BioVeL Portal*

[*http://www.gbif.org/resource/80922*](http://www.gbif.org/resource/80922)

**Pointers to specific help information, with reference to Figure 1**

*
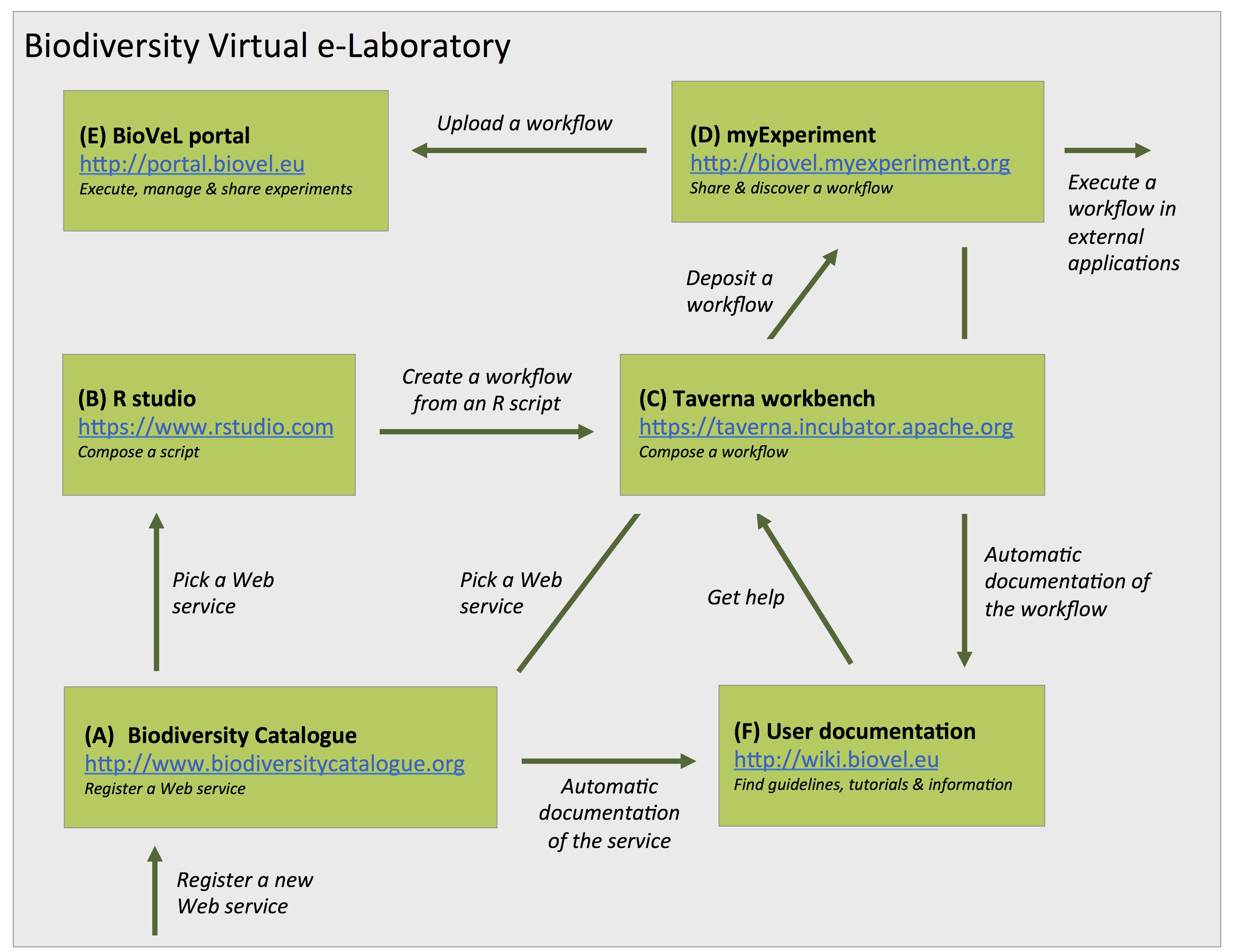
*

**Figure 1: Biodiversity Virtual Laboratory (BioVeL) is a software environment that assists scientists in collecting, organising, and sharing data processing and analysis tasks in biodiversity and ecological research. The main components of the platform are: (A) the Biodiversity Catalogue (a library with well-annotated data and analysis services); (B) the environment, such as RStudio for creating R programs; (C) the workbench for assembling data access and analysis pipelines; (D) the myExperiment workflow library that stores existing workflows; (E) the BioVeL Portal that allows researchers and collaborators to execute and share workflows; and (F) the documentation wiki. Infrastructure is indicated in bold, while processes related to research activities are indicated in italics.**

***Component (A): Register Web services and the Biodiversity Catalogue***

*General information on Web services*

[*https://www.biocatalogue.org/wiki/doku.php?id=public:help:general_info_on_web_services*](https://www.biocatalogue.org/wiki/doku.php?id=public:help:general_info_on_web_services)

*Best practice guidelines for developing Web services*

[*https://wiki.biovel.eu/display/doc/Creating+Web+Services+for+BioVeL*](https://wiki.biovel.eu/display/doc/Creating+Web+Services+for+BioVeL)

[*http://dev.mygrid.org.uk/wiki/display/scrap/Web+services+guidelines*](http://dev.mygrid.org.uk/wiki/display/scrap/Web+services+guidelines)

*Getting started on Biodiversity Catalogue*

[*https://wiki.biovel.eu/display/doc/BiodiversityCatalogue*](https://wiki.biovel.eu/display/doc/BiodiversityCatalogue)

*How to register a service on Biodiversity Catalogue*

[*https://wiki.biovel.eu/display/doc/BiodiversityCatalogue#BiodiversityCatalogue-RegisterServices*](https://wiki.biovel.eu/display/doc/BiodiversityCatalogue#BiodiversityCatalogue-RegisterServices)

***Component (B, C): Composing scripts and workflows with Web services***

*How to find a service on Biodiversity Catalogue*

[*https://wiki.biovel.eu/display/doc/BiodiversityCatalogue#BiodiversityCatalogue-ServiceDiscovery*](https://wiki.biovel.eu/display/doc/BiodiversityCatalogue#BiodiversityCatalogue-ServiceDiscovery)

*How to create an R script that calls a Web service*

[*https://github.com/BioVeL/biovel-R-client*](https://github.com/BioVeL/biovel-R-client)

*How to create and document a workflow*

[*https://wiki.biovel.eu/display/doc/Taverna+Workbench*](https://wiki.biovel.eu/display/doc/Taverna+Workbench)

[*https://wiki.biovel.eu/display/doc/Creating+Taverna+2+Workflows*](https://wiki.biovel.eu/display/doc/Creating+Taverna+2+Workflows)

*How to create interaction pages*

[*https://wiki.biovel.eu/display/doc/Creating+Interaction+Pages*](https://wiki.biovel.eu/display/doc/Creating+Interaction+Pages)

***Component (D): Share and discover workflows on myExperiment***

*How to execute and share workflows with Taverna*

[*http://www.slideshare.net/mygrid/2014-taverna-tutorial-myexperiment*](http://www.slideshare.net/mygrid/2014-taverna-tutorial-myexperiment)

*How to execute workflows in external applications*

[*https://wiki.biovel.eu/display/doc/Integrating+Taverna+Player++into+Scratchpads*](https://wiki.biovel.eu/display/doc/Integrating+Taverna+Player++into+Scratchpads)

*How to parallelise the calculations in a workflow*

[*http://dev.mygrid.org.uk/wiki/display/tav250/Advanced+workflow+configuration*](http://dev.mygrid.org.uk/wiki/display/tav250/Advanced+workflow+configuration)

[*http://www.myexperiment.org/files/1068.html*](http://www.myexperiment.org/files/1068.html) *(Powerpoint tutorial)*

***Component (E): How to upload, execute, and share workflows and results on the BioVeL portal***

*Introduction to the BioVeL Portal*

[*https://wiki.biovel.eu/display/doc/BioVeL+Portal*](https://wiki.biovel.eu/display/doc/BioVeL+Portal)

*How to access the BioVeL portal*

[*https://wiki.biovel.eu/display/doc/Accessing+the+BioVeL+Portal*](https://wiki.biovel.eu/display/doc/Accessing+the+BioVeL+Portal)

*How to inspect a workflow on the BioVeL portal* [*https://wiki.biovel.eu/display/doc/Inspecting+a+Workflow+on+the+BioVeL+Portal*](https://wiki.biovel.eu/display/doc/Inspecting+a+Workflow+on+the+BioVeL+Portal)

*How to run a workflow on the BioVeL Portal*

[*https://wiki.biovel.eu/display/doc/Running+a+Workflow+on+the+BioVeL+Portal*](https://wiki.biovel.eu/display/doc/Running+a+Workflow+on+the+BioVeL+Portal)

*Functionalities of registered users*

[*https://wiki.biovel.eu/display/doc/Functionalities+for+Registered+Users*](https://wiki.biovel.eu/display/doc/Functionalities+for+Registered+Users)

***Component (F): How to find information and get help***

*BioVel wiki*

[*https://wiki.biovel.eu/display/doc/BioVeL+Wiki*](https://wiki.biovel.eu/display/doc/BioVeL+Wiki)

*If you cannot find information you think should be in the Wiki, or something is not sufficiently clearly explained, please contact us by email to* [*support@biovel.eu*](mailto:support@biovel.eu)
