# Supplementary material for: BioVeL: a virtual laboratory for data analysis and modelling in biodiversity science and ecology
Source: BMC Ecol. 2016 Oct 20;16:49. doi: 10.1186/s12898-016-0103-y (PMC5073428; doi:10.1186/s12898-016-0103-y)
Supplement: Supplementary file 2 — Additional file 2: Table S1. Service groups and capabilities for processing and analysis in biodiversity science. [file 12898_2016_103_MOESM2_ESM.docx]

**Supplementary Information**

Table 1 (detailed): Service groups and capabilities for processing and analysis in biodiversity science

| Service set | Capabilities (Web services) |
| --- | --- |
| General purpose, including mapping and visualization | General-purpose capabilities needed in many situations, such as for:   - Interactive visualization of spatio-temporal data e.g., occurrence data; - Execution of R-scripts embedded as steps in workflows; - Temporary workspace for data file movements between services.   Services include:  [**BioSTIF interaction service for visualization of occurrence data**](https://www.biodiversitycatalogue.org/services/7): A web map application designed for interactive visualization of spatio-temporal data, such as occurrence data. It allows comparison of different data sources having at least one spatial attribute with a geographic coordinate (point). Data can be manipulated using geospatial, temporal and table-based filters.  [**BioSTIF upload service**](https://www.biodiversitycatalogue.org/services/6): Allows to store data into a server where it can be used by the BioSTIF interaction service.  **Rserve &** [**OpenCPU**](https://www.biodiversitycatalogue.org/services/32) **services**: Provide environments for remote execution of R scripts embedded in workflows. |
| Ecological niche modelling | Built up from the existing openModeller web service [34] to offer a wide range of algorithms and modelling procedures integrated with geospatial management of environmental data, enabling researchers to create, test, and project ecological niche models (ENM).  Services include:  [**openModeller web service**](https://www.biodiversitycatalogue.org/services/37): Based on the openModeller software suite from CRIA, the purpose of the openModeller web service is to allow ecological niche models to be created, tested and projected into different environmental scenarios over the World-wide Web. |
| Ecosystem modelling | A basic toolbox for studies of carbon sequestration and ecosystem function. It includes data-model integration and calibration services, model testing and Monte Carlo Experiment services, ecosystem valuation services, and bioclimatic services. Two variants of the model are implemented: Biome-BGC v4.1.1 MPI and Biome-BGCMuSo v3.0.  Services include:  [**Get local meteorological datasets and/or meteorological scenarios**](https://www.biodiversitycatalogue.org/services/58): Provides a local daily meteorological dataset for modelling at a given location. Data is currently retrieved from the Open Database FOR ClimatE Change-Related Impact Sudies in CEntral Europe (FORESEE meteorological database), that covers the 1951-2100 time period and contains observed and projected daily maximum/minimum temperature and precipitation variables for Central Europe.  [**Biome-BGC Monte Carlo Experiment**](http://purl.ox.ac.uk/workflow/myexp-3620.4): Runs Biome-BGC model in a high number of instances with randomly sampled input parameter sets.  [**Biome-BGC Sensitivity Analysis**](http://purl.ox.ac.uk/workflow/myexp-4490.1): Gets the results of a Monte Carlo Experiment executed previously, then calculates parameter sensitivity of selected output variables.  [**Biome-BGC Generalized Likelihood Uncertainty Estimation (GLUE)**](http://purl.ox.ac.uk/workflow/myexp-4481.2): Provides calibration of model settings with field measurement datasets, based on comparison and evaluation (calculating likelihoods) of the results of a Monte Carlo Experiment and measured data sets.  [**Biome-BGC CARBON**](https://www.biodiversitycatalogue.org/services/50): Runs a single Biome-BGC model version instance, allowing model versions, parameter sets (model settings, output settings) and input datasets to be selected (ecophysiology, site characteristics, meteorology, site datasets).  [**Biome-BGC Ecosystem Service Indicators**](https://www.biodiversitycatalogue.org/services/49): Runs a single Biome-BGC model instance and provides a set of ecosystem service indicators. These include: annual wood increment, yearly production of grasslands or croplands, total average carbon stock, annual evapotranspiration, damping of ecosystem daily water outflow, living and dead biomass protecting the soil against erosion, litter and coarse woody debris decomposition rate, and humification rate in the soil. |
| Metagenomics | A basic set of services for studying community structure and function from metagenomic ecological datasets. It includes services for geo-referenced annotation, metadata services, taxonomic binning and classification services, metagenomic traits services, and services for multivariate analysis.  Services include:  [**Metagenomics Traits Services (MTS)**](https://www.biodiversitycatalogue.org/services/64): Delivers ecologically interesting traits information of bacterial communities as observed by high-throughput metagenomic DNA sequencing. Multiple operations as follows:  **MTS Functional content**: Returns the list of functional annotations per metagenomic sample. The current version supports PFAM annotations based on PFAM database version 27.  **MTS Amino acid content**: Calculates the amino acid composition of the predicted proteins based on 'cusp' bundled in the EMBOSS package.  **MTS Codon usage calculation**: Calculates the codon usage of the predicted proteins based on 'cusp' bundled in the EMBOSS package.  **MTS GC variance**: Calculates normal GC-variance based on 1000-2000 basepair sliding windows; for metagenomes the sliding window is each read.  **MTS Di-nucleotide odds ratio**: Retrieves the di-nucleotide-odds ratio of a given sequence set.  **MTS Taxonomic content**: Calculates the occurrences of Bacteria and Archaea based on 16S rDNA predictions and taxonomic classification by ARB/SINA based on the SILVA database.  **MTS Simple traits**: Gives basic information about a metagenomic sample (id, description geographic origin) and traits (environment, environmental ontology classification, GC Content, GC Variance, number of genes, total of bases (MB), number of reads, AB Ratio, Perc Tf, Perc classified).  **MTS All**: Returns all simple traits of all metagenomic samples from Metagnomic Traits Database.  [**(BioMaS) Bioinformatic analysis of Metagenomic ampliconS)**](https://www.biodiversitycatalogue.org/services/71): Is a bioinformatic pipeline supporting biomolecular researchers to carry out taxonomic studies of environmental microbial communities by a completely automated workflow, comprehensive of all the fundamental steps, from raw sequence data arrangement to final taxonomic identification, that are absolutely required in a typical Meta-barcoding HTS-based experiment. In its current version, BioMaS allows analysis of both bacterial and fungal environments starting directly from the output of the Illumina platforms. |
| Phylogenetics | Services to enable DNA sequence mining and alignment, core phylogenetic inference, tree visualization, and phylogenetic community structure, for broad use in evolutionary and ecological studies.  Services include:  [**Phylogenetics_AT_JST**](https://www.biodiversitycatalogue.org/services/31): Is a collection of methods (services) to define model, perform, test and use phylogenetic inference in downstream applications. Multiple services as follows:  **MadTranslator45**: Translates nucleotide sequences using a user defined genetic code/s and reading frame/s and cuts the translated sequence at each stop codon.  **HMMSearchAlign_Pmerge**: Performs a search using amino acid sequences as queries against a local mirror of PFAM database (updated every 3 months) and performs a multiple sequence alignment of either single or multiple protein domains coding sequences.  **MultiSingDomAlign**: Performs either single or multiple domain protein coding DNA sequences.  **PartitionFinder**: Using PartitionFinder and uploading annotation of partitions and multifasta MSA’s the service indicates best partitioned models for the data, assuming a given range of possible partition models selected using AIC/AICc/BIC approach.  **MrBayes_16CPUs**: Executes a parallel version MrBayes 3.2.1 to obtain a Bayesian phylogenetic inference.  **Re_MrBayes_16CPUs**: Re-executes a parallel version MrBayes 3.2.1 to obtain a Bayesian phylogenetic inference using as input the output of previous run.  **RAxML_partition**: Executes a thread version RAxML allowing to define a partitioned model with option -q and -M.  **GeoKS_xml**: Compares the posterior distribution of trees in pairwise fashion across all the independent runs of MrBayes and calculates the overall probability of convergence using a Fisher procedure to compose the different Kolmogorov-Smirnov p-values.  **MrBayesPPtest**: Performs a comparison between observed complexity of the data and distribution of simulated data from the posterior distribution (i.e. posterior predictive test).  **ConsensusTree**: Builds a consensus tree over the posterior distribution tree and computes branch length average only among bipartition present in the consensus topology.  **PhyloH**: Identifies a lineage over a tree that contributes the most to the phylogenetic differentiation (i.e. phylogenetic beta diversity) across groups of samples.  [**SUPERSMART (Self-Updating Platform for Estimating Rates of Speciation and Migration, Ages and Relationships of Taxa)**](https://www.biodiversitycatalogue.org/services/78): Is a pipeline analytical environment for large-scale phylogenetic data mining, taxonomic name resolution, tree inference and fossil-based tree calibration. Like the Phylogenetics_AT_JST service above, it consists of a collection of methods.  [**NeXML parser and coder**](https://www.biodiversitycatalogue.org/services/70): Is a service that allow users to enrich and combine datasets encoded in standard phylogenetic data formats (e.g., Newick, NEXUS, etc.) into an integrated NeXML representation, and to extract subsets of the data from NeXML documents. |
| Population modelling | Services for demographic data and their integration into matrix projection models and integral projection models (MPM, IPM). For [**MPM**](http://purl.ox.ac.uk/researchobj/myexp-483), services are based on the popbio and popdemo R packages. For [**IPM**](http://purl.ox.ac.uk/researchobj/myexp-482), services are based on the IPMpack R package. |
| Taxonomy | Services for taxonomic name resolution, checklists and classification, and species occurrence data retrieval.  Services include:  [**Catalogue of Life checklist web service**](https://www.biodiversitycatalogue.org/services/24): The Catalogue of Life is a checklist of scientific name species. The checklist web service can be used to expand given names into associated names (i.e., accepted name, synonyms, etc.). It is provided by Naturalis Biodiversity Center, The Netherlands.  [**BGBM CoL checklist web service**](https://www.biodiversitycatalogue.org/services/17): Offered by the Botanic Gardens and Botanical Museum, Berlin, providing access to various checklists, including the Catalogue of Life and PESI.  [**GBIF occurrence web service**](https://www.biodiversitycatalogue.org/services/92): GBIF makes available biodiversity data that are shared by hundreds of data publishers from around the world. The occurrence retrieval web service is used to retrieve species occurrence records for given scientific names.  [**openRefine data cleaning web service**](https://www.biodiversitycatalogue.org/services/65): Provides a Web service interface to the open source openRefine tool for working with messy data. |
