# Supplementary material for: BioVeL: a virtual laboratory for data analysis and modelling in biodiversity science and ecology
Source: BMC Ecol. 2016 Oct 20;16:49. doi: 10.1186/s12898-016-0103-y (PMC5073428; doi:10.1186/s12898-016-0103-y)
Supplement: Supplementary file 3 — Additional file 3: Table S2. Workflows for biodiversity science. [file 12898_2016_103_MOESM3_ESM.docx]

**Supplementary Information**

**Table 2 (detailed): Workflows for biodiversity science**

Ctrl+Click workflow name below to jump to relevant part of the table

For help and assistance beyond that available via the documentation links, contact [support@biovel.eu](mailto:support@biovel.eu).

[Data Refinement Workflow (DRW) 1](#_Toc435178922)

[Generic Ecological Niche Modelling workflow (ENM) 3](#_Toc435178923)

[BioClim workflow 4](#_Toc435178924)

[ENM statistical difference workflows (ESW) 5](#_Toc435178925)

[Matrix Population Modelling collection (MPM) 6](#_Toc435178926)

[Integral Projection Models workflow (IPM) 8](#_Toc435178927)

[Biome-BGC CARBON workflow 9](#_Toc435178928)

[Biome-BGC Ecosystem Service Indicators workflow (ESI) 11](#_Toc435178929)

[Biome-BGC Sensitivity Analysis workflows (SA) 12](#_Toc435178930)

[Biome-BGC Generalized Likelihood Uncertainty Estimation workflow (GLUE) 13](#_Toc435178931)

[Microbial Metagenomic Trait Statistical Analysis Workflow (MMT) 14](#_Toc435178932)

[Bioinformatic analysis of Metagenomic ampliconS workflow (BioMaS) 15](#_Toc435178933)

[Phylogenetic workflows 16](#_Toc435178934)

| Workflow | **Data Refinement Workflow (DRW)** |
| --- | --- |
| Family | Data preparation |
| What is it for? | The Data refinement workflow is for preparing observational and specimen data sets for use in scientific analyses such as: species distribution analysis, species richness and diversity studies, species occurrence studies, historical analysis, and other spatio-temporal analyses. It can be used to validate taxonomic data sets (evaluating different name usages, including species concepts, misspellings and other incongruences) and to retrieve, integrate, clean, and refine species occurrence records as well as associated quantitative information (e.g., biomass). |
| Who is it for? | Scientists and biodiversity managers who need to integrate their own collection, observational, and taxonomic name data with data from distributed biodiversity services. |
| How does it work? | The workflow consists of three sub-workflows for:   1. Data integration, 2. Data cleaning, refinement, and filtering, and 3. Geographic and/or temporal selection.   The sub-workflows can be executed and repeated in arbitrary order. Being based on standard data formats for input, output, and internal processing, the workflow can be left and re-visited at any point of execution. |
| Expected results | Enriched, harmonized, and/or filtered datasets, in csv and Darwin core format. |
| Links to workflows and user’s documentation | purl: <http://purl.ox.ac.uk/workflow/myexp-2874.13>  myExperiment: <http://www.myexperiment.org/workflows/2874.html>  Portal: <https://portal.biovel.eu/workflows/641>  Documentation: <https://wiki.biovel.eu/x/KYAnAQ>  In addition to regular documentation, a training manual specifically designed for use with GBIF data: <http://www.gbif.org/resource/81239>. |
| Publications | Leidenberger et al. (2013) Mapping present and future potential distribution patterns for a meso-grazer guild in the Baltic Sea. doi:[10.1111/jbi.12395](http://dx.doi.org/10.1111/jbi.12395).  Leidenberger et al. (2015) Evaluating the potential of ecological niche modelling as a component in marine non-indigenous species risk assessments. doi:[10.1016/j.marpolbul.2015.04.033](http://dx.doi.org/10.1016/j.marpolbul.2015.04.033).  Mathew C, et al. (2014) A semi-automated workflow for biodiversity data retrieval, cleaning, and quality control. doi:[10.3897/BDJ.2.e4221](http://dx.doi.org/10.3897/BDJ.2.e4221). |
| Examples of use | A benchmark comparison exercise was conducted on two datasets with:   1. Historical dataset with 7,400 species observation data from the Swedish West coast, collected between 1921–1938; and, 2. Recent dataset with 4,100 species observation data from the Swedish West coast, collected between 2003–2009   With a “traditional” Excel-based data refinement technique, it took 38½ hours to clean these datasets. With the BioVeL DRW, it took 4¼ hours to obtain the same results.  A further example, of a phyloclimatic analysis of Cucumber integrating the analytical features of various (DRW, ENM, Phylogenetics) workflows, can be found in the repository of the BEsFE 2014 meeting in Lille: <http://bit.do/PhyloClimatic>. |
| Partners and *contributors | [Botanic Garden and Botanical Museum Berlin-Dahlem](http://www.bgbm.org/en) (BGBM), Freie Universität Berlin, Germany  [Fraunhofer Institute for Intelligent Analysis and Information](http://www.iais.fraunhofer.de/) (Fraunhofer IAIS), Germany  [University of Eastern Finland](https://www.uef.fi/en) (UEF), Finland |

[Back to top](#T2expTOP)

| Workflow | **Generic Ecological Niche Modelling workflow (ENM)** |
| --- | --- |
| Family | Ecological Niche Modelling (ENM) |
| What is it for? | The ENM workflow family offers a framework to study geographic distributions of many species under various climatic scenarios. ENM uses associations between known species occurrences and environmental variables to calculate areas of habitat suitability for a species using statistical correlation models.  Studies of species adaptations to climate change, dynamic modelling of ecologically related species, and identification of regions with accumulated risk for invasion, potential for restoration, or need for protection. |
| Who is it for? | Researchers in ecology and evolution analyzing biogeographic patterns over large spatial, temporal, environmental and taxonomic scales. |
| How does it work? | This workflow takes as input a file containing species occurrence points to create an ecological niche model with the openModeller Web Service algorithm, environmental layers and geographic area are selected during the workflow. The model is tested (internal test and optional cross validation external test) and then projected one or more times. All points from the input file are used to create a single model, even if there are differences in the scientific names. Model projections can be downloaded from the links in the workflow output. |
| Expected results | Potential distribution maps for a large number of species and parameter settings. |
| Links to workflows and user’s documentation | purl: <http://purl.ox.ac.uk/workflow/myexp-3355.20>  myExperiment: <http://www.myexperiment.org/workflows/3355.html>  Portal: <https://portal.biovel.eu/workflows/440>  Documentation: <https://wiki.biovel.eu/x/ooSk> |
| Publications | Leidenberger et al. (2013) Mapping present and future potential distribution patterns for a meso-grazer guild in the Baltic Sea. doi:[10.1111/jbi.12395](http://dx.doi.org/10.1111/jbi.12395).  Leidenberger et al. (2015) Evaluating the potential of ecological niche modelling as a component in marine non-indigenous species risk assessments. doi:[10.1016/j.marpolbul.2015.04.033](http://dx.doi.org/10.1016/j.marpolbul.2015.04.033). |
| Example of use | Modelling European forest insect pests - see other supplementary information.  Potential changes in the distribution of major European forest insect pests were analyzed for various climate change scenarios. This involved first modelling the historical and current distribution of both host trees and the insects. Then, likely changes in the distribution of the host trees were modelled and this information was used as the environmental inputs for modelling the future potential distribution of the insects. Both GBIF occurrences and actual forest damage reports from the EFI-Alterra Database of Forest Disturbances in Europe were used as data sources. The predictions showed that the damage by most major insect pests will spread about 500 km towards north east by the year 2050, but much uncertainty lies in how far the host trees will actually be capable of moving or will be planted. |
| Partners and *contributors | [Centro de Referência em Informação Ambiental](http://www.cria.org.br/) (CRIA), Brazil  [Fraunhofer Institute for Intelligent Analysis and Information](http://www.iais.fraunhofer.de/) (Fraunhofer IAIS), Germany  [University of Manchester](http://www.manchester.ac.uk/), UK  [Cardiff University](http://www.cardiff.ac.uk/), UK |

[Back to top](#T2expTOP)

| Workflow | **BioClim workflow** |
| --- | --- |
| Family | Ecological Niche Modelling (ENM) |
| What is it for? | The BioClim workflow is usually run before the ENM workflow to select environmentally unique points from a specified set of points and to calculate the corresponding environmental envelopes (minimum/maximum) for the given environmental variables. It is a variation of the ENM workflow, described above. With this workflow you can filter a set of species occurrences for environmentally unique points. This procedure avoids passing redundant information to niche modelling algorithms later. Besides filtering the points, the workflow generates a BioClim model to calculate the environmental range for each variable. |
| Who is it for? | Users of ENM workflow, wishing to pre-process and prepare their input data for niche modelling. |
| How does it work? | It creates an ecological niche model with the openModeller Web Service using the Bioclim algorithm. Environmental layers and geographic mask are selected during the workflow. Points are filtered so that only environmentally unique points are used to create the model. |
| Expected results | Filtered set of points (environmentally unique) and Bioclim model where environmental envelopes can be found. |
| Links to workflows and user’s documentation | purl: [<http://purl.ox.ac.uk/workflow/myexp-3725.2>](http://purl.ox.ac.uk/workflow/myexp-3725.2)  myExperiment: <http://www.myexperiment.org/workflows/3725.html>  Portal: <https://portal.biovel.eu/workflows/443>  Documentation: <https://wiki.biovel.eu/x/X4Gz> |
| Publications | Leidenberger et al. (2013) Mapping present and future potential distribution patterns for a meso-grazer guild in the Baltic Sea. doi:[10.1111/jbi.12395](http://dx.doi.org/10.1111/jbi.12395).  Leidenberger et al. (2015) Evaluating the potential of ecological niche modelling as a component in marine non-indigenous species risk assessments. doi:[10.1016/j.marpolbul.2015.04.033](http://dx.doi.org/10.1016/j.marpolbul.2015.04.033). |
| Example of use | The BioClim workflow can be used to obtain a subset of environmentally unique occurrence points from a larger set of occurrence data. This can be useful in situations where large numbers of observations in a particular geographic/environmentally similar locality may have the effect of skewing the modelling of the species distribution.  The workflow can also be used to gain a useful overview of the environmental ranges where the species can be found. |
| Partners and *contributors | [Centro de Referência em Informação Ambiental](http://www.cria.org.br/) (CRIA), Brazil  [University of Gothenburg](http://www.gu.se/), Sweden |

[Back to top](#T2expTOP)

| Workflow | **ENM statistical difference workflows (ESW)** |
| --- | --- |
| Family | Ecological Niche Modelling (ENM) |
| What is it for? | The ENM Statistical Difference (DIFF) and Statistical Stack (STACK) workflows are for post-processing outputs from the ENM workflow. They perform raster layer comparisons, computing the extent and intensity of changes in species’ potential distributions between two scenarios or model projects (DIFF) or aggregation of multiple projections (STACK), or computation of the shift in centrepoints between two scenarios. |
| Who is it for? | Users of the ENM workflow. |
| How does it work? | ESW DIFF workflow computes extent, direction, intensity of change in species potential distribution, including change in the centre point of the distribution by computing differences between two different projections of the ecological niche model e.g., present day and future time.  ESW STACK workflow computes extent, intensity, and a cumulated potential species distribution by computing the average sum from multiple ecological niche models e.g., for several related species. |
| Expected results | Difference maps, stack maps (average sum layer from multiple raster layers), shift maps |
| Links to workflows and user’s documentation | ESW DIFF  purl: <http://purl.ox.ac.uk/workflow/myexp-3959.2>  myExperiment: <http://www.myexperiment.org/workflows/3959.html>  Portal: <https://portal.biovel.eu/workflows/442>  Documentation: <https://wiki.biovel.eu/x/AgD1>  ESW STACK  purl: <http://purl.ox.ac.uk/workflow/myexp-3856.3>  myExperiment: <http://www.myexperiment.org/workflows/3856.html>  Portal: <https://portal.biovel.eu/workflows/70>  Documentation: <https://wiki.biovel.eu/x/54L7> |
| Publications | Leidenberger et al. (2013) Mapping present and future potential distribution patterns for a meso-grazer guild in the Baltic Sea. doi:[10.1111/jbi.12395](http://dx.doi.org/10.1111/jbi.12395). |
| Example of use | A research group at the Center for Marine Evolutionary Biology (CEMEB), University of Gothenburg, used BioVeL workflows to study present and future distribution patterns of endemic Baltic Sea species that may be threatened by climate change. The study uses ecological niche modelling techniques, including the ESW DIFF and ESW STACK workflows to study potential distribution for a food web consisting of a guild of meso-grazers (*Idotea* spp.), their host algae (*Fucus vesiculosus* and *Fucus radicans*) and their fish predator (*Gasterosteus aculeatus*). The study shows that a north-eastern shift of *I. balthica* and *I. chelipes* into the distribution area of the endemic alga *F. radicans* in the Baltic Sea may result in increased grazing pressure and extinction risk for the species. |
| Partners and *contributors | [Centro de Referência em Informação Ambiental](http://www.cria.org.br/) (CRIA), Brazil  [University of Gothenburg](http://www.gu.se/), Sweden |

[Back to top](#T2expTOP)

| Workflow | **Matrix Population Modelling collection (MPM)** |
| --- | --- |
| Family | Population Modelling |
| What is it for? | Matrix population models (and integral projection models, below) are among the most widely used tools to model the dynamics of plants and wildlife populations. They play a central role in ecology, evolution and conservation biology.  The Matrix Population Model (MPM) collection comprises 24 workflows that together provide a complete environment for creating a stage-matrix or series of matrices with no density dependence, and then to perform several analyses on it/them for research and studies based on population modelling. This collection allows:  1. Age-specific survival analysis  2. Bootstrap of observed census transitions  3. Calculate and plot the abundances over the years  4. Calculate quasi-extinction threshold  5. Cohen cumulative distance  6. Create log-log plots of variance vs. sensitivity and cV vs. elasticity  in matrix elements  7. Damping ratio  8. Eigen analysis  9. Elasticity and Sensitivity of the Vital rates  10. Generation time (T)  11. Keyfitz’s delta  12. Life Table Response Experiment fixed design Place Effect  13. Life Table Response Experiment Year Effect in One Location or Place  14. Life Table Response Experiments year effect for multiple places  15. Matrix Population Model analysis v12  16. Matrix Population Model construction  17. Matrix Population Model construction and analysis v20  18. Mean Matrix  19. Net reproductive rate (Ro)  20. Parametric Bootstrap or Resample a projection matrix Workflow  21. Simulate stochastic growth from a sequence of matrices  22. Stage vectors, Survival and Growth rate curves (demography)  23. Transient Dynamics  24. Variance matrix |
| Who is it for? | Biology, forestry and agriculture students, scientists and biodiversity resources managers. |
| How does it work? | These workflows are based mainly on the popbio and popdemo R packages.  For popbio:  <http://cran.r-project.org/web/packages/popbio/index.html>  <http://cran.r-project.org/web/packages/popbio/popbio.pdf>  <http://www.jstatsoft.org/v22/i11/paper>  For popdemo:  <http://cran.r-project.org/web/packages/popdemo/index.html>  <http://cran.r-project.org/web/packages/popdemo/popdemo.pdf>  <http://onlinelibrary.wiley.com/doi/10.1111/j.2041-210X.2012.00222.x/abstract> |
| Expected results | Numerical results (.csv and .txt documents and graphs .jpg and .png) |
| Links to workflows and user’s documentation | purl: <http://purl.ox.ac.uk/researchobj/myexp-483>  myExperiment: <http://www.myexperiment.org/packs/483.html>  Each workflow in the collection is also available separately. For listing see: <http://www.myexperiment.org/users/20384/packs>  Portal: <https://portal.biovel.eu/workflows/596>  Documentation: <https://wiki.biovel.eu/x/WgB0> |
| Example of use | From a study of the relationship between lambda (population growth rate), calculated from projection matrices, and the time since disturbance of the vegetation in the habitat of *Gentiana pneumonanthe* (Marsh gentian) it became clear what managers needed to do to get this rare and protected species to recover in their area.  The study showed that in the early stages after a disturbance that opens up the vegetation structure, there is ample opportunity for seedling recruitment and the populations grow rapidly. After some years, the vegetation structure has closed so much by natural succession that light competition prevents most of the seeds from germinating and new seedlings cannot establish. Because of this, population growth rates (lambda's) drop and the population starts to decline. The decline is slow because adult plants are relatively long-lived. Population extinction would occur after 40-45 years without new disturbance; for example by opening up the vegetation by sod removal, mowing or sheep or cattle grazing. |
| Partners and *contributors | University of Amsterdam, [Institute of Biodiversity and Ecosystem Dynamics](http://ibed.uva.nl/), The Netherlands  [Cardiff University](http://www.cardiff.ac.uk/), UK  *[cOMPaDRE database](http://www.compadre-db.org/) is supported by the Laboratory on Evolutionary Biodemography, Max Planck institute for Demographic Research, Rostock, Germany. |

[Back to top](#T2expTOP)

| Workflow | **Integral Projection Models workflow (IPM)** |
| --- | --- |
| Family | Population Modelling |
| What is it for? | Integral projection models (and matrix population models, above) are among the most widely used tools to model the dynamics of plants and wildlife populations. They play a central role in ecology, evolution and conservation biology.  The Integral Projection Model (IPM) workflow provides an environment to create and test the IPM, and allows users to perform several analyses on that for research and studies based on population modelling. IPMs are related to classic matrix models (see MPM workflow above) but IPMs are more appropriate for modelling structured populations when the variable describing an individuals’ demography is continuous (e.g., size, weight, etc.) |
| Who is it for? | Biology, forestry and agriculture students, scientists and biodiversity resources managers. |
| How does it work? | This workflow is based mainly on the IPMpack R package.  <http://ipmpack.r-forge.r-project.org/>  <http://cran.r-project.org/web/packages/iPMpack/index.html>  <http://cran.r-project.org/web/packages/iPMpack/iPMpack.pdf>  [http://cran.r-project.org/web/packages/iPMpack/vignettes/iPMpack_ Vignette.pdf](http://cran.r-project.org/web/packages/iPMpack/vignettes/iPMpack_%20Vignette.pdf)  <http://onlinelibrary.wiley.com/doi/10.1111/2041-210x.12001/abstract> |
| Expected results | Numerical results (.csv and .txt documents and graphs .jpg and .png) |
| Links to workflows and user’s documentation | purl: <http://purl.ox.ac.uk/researchobj/myexp-482>  myExperiment: <http://www.myexperiment.org/packs/482.html>  Portal: <https://portal.biovel.eu/workflows/599>  Documentation: <https://wiki.biovel.eu/x/mYOk> |
| Example of use | Using the IPM workflow the user can expect to construct an IPM matrix and analyse it for sensitivity, elasticity, vital rates sensitivity and elasticity, survivorship, and mean life expectancy and passage time. See, for example: Merow et al (2014) Advancing population ecology with integral projection models: a practical guide. doi: [10.1111/2041-210X.12146](http://dx.doi.org/10.1111/2041-210X.12146) |
| Partners and *contributors | University of Amsterdam, [Institute of Biodiversity and Ecosystem Dynamics](http://ibed.uva.nl/), The Netherlands  [Cardiff University](http://www.cardiff.ac.uk/), UK  *Roberto Salguero-Gómez, University of Queensland, Australia. |

[Back to top](#T2expTOP)

| Workflow | **Biome-BGC CARBON workflow** |
| --- | --- |
| Family | Ecosystem Modelling |
| What is it for? | A meteorology driven biogeochemical ecosystem model, Biome-BGC simulates the carbon, water and nitrogen fluxes and pools of terrestrial ecosystems. The Biome-BGCMuSo variant performs more realistic simulations in terms of soil hydrology, and improved ecosystem management options. The family of workflows supports the use of Biome-BGC for ecosystem modelling applications.  This workflow supports studies of the impact of climate change or management scenarios on terrestrial ecosystems through models under different environmental conditions. |
| Who is it for? | Scientists interested in carbon sequestration or carbon, nitrogen and water cycling simulations of various terrestrial ecosystems: i.e. grass- lands, shrublands, forests, cultivated lands |
| How does it work? | The BBGC CARBON workflow executes a single simulation run at a given geographic location under distinctive environmental circumstances, and for a specified time-span of years, past and/or future climate scenarios and management options.  Preparation of the various inputs and settings is required first, then a simulation run can be performed. A simulation requires specific inputs like:   - Local daily meteorology input dataset - A number of general ecophysiological constants as essential parameters - Local site/soil parameters - Annual atmospheric CO2 concentration (optional) - Annual nitrogen deposition (optional) - Annual mortality scenarios (optional only for MuSo) - Annual groundwater scenarios (optional only for MuSo) - Annual management scenarios (optional only for MuSo) - Output control settings of Biome-BGC variables - Spinup and normal initialization settings.   A run consists of a so-called spinup (“warming up”) phase and pipelined normal (“productive”) simulation phases. The Biome-BGC Project Database & Management System (BBGCDB) supports users to manage various Biome-BGC ecosystem modelling investigations embedded in Taverna workflow environment on BioVeL Portal. |
| Expected results | Structured time-series (daily, monthly and/or annual) output text files, zipped and attached to the BBGCDB project record. BBGCDB project ID, URL and standard annual summary output result from the workflow. |
| Links to workflows and user’s documentation | purl: <http://purl.ox.ac.uk/researchobj/myexp-687> (whole family pack)  myExperiment: <http://www.myexperiment.org/workflows/3618>  Portal: <https://portal.biovel.eu/workflows/81>  Documentation: <https://wiki.biovel.eu/x/DQEtAQ> |
| Example of use | Comparing the Net Ecosystem Exchange (NEE), Leaf area index (LAI), Gross Primary Production (GPP) and Respiration (Reco) time-line of Biome-BGCMuSo and Biome-BGC MPI versions with the same parametrization from 2006 to 2013 at Hegyhátsál, Hungary shows that both models underestimate the variability of the carbon fluxes. The original model (MPI) performs better, because calibration was tuned to this version. Further improvements of MuSo model were made. This shows that new calibration is needed for new model construction. |
| Partners and *contributors | [Centre for Ecological Research](http://www.okologia.mta.hu/en/node/2), Academy of Sciences (MTA ÖK), Hungary  [University of Eastern Finland](http://www.uef.fi/)  *[Croatian Forest Research Institute](http://www.sumins.hr/en/), Croatia  *[Department of Meteorology](http://nimbus.elte.hu/index-en.html), Eötvös Loránd University, Hungary  *[Finnish Environment Institute (SYKE)](http://www.syke.fi/en-US), Finland  *[Laboratory of Parallel and Distributed Systems (MTA SZTAKI)](https://www.lpds.sztaki.hu/), Hungary  *[National Forest Centre](http://www.nlcsk.sk/), Forest Research Institute, Slovak Republic  *[University of Pécs](http://pte.hu/english), Hungary  *[Szent István University](http://sziu.hu/), Hungary |

[Back to top](#T2expTOP)

| Workflow | **Biome-BGC Ecosystem Service Indicators workflow (ESI)** |
| --- | --- |
| Family | Ecosystem Modelling |
| What is it for? | A meteorology driven biogeochemical ecosystem model, Biome-BGC simulates the carbon, water and nitrogen fluxes and pools of terrestrial ecosystems. The Biome-BGCMuSo variant performs more realistic simulations in terms of soil hydrology, and improved ecosystem management options. The family of workflows supports the use of Biome-BGC for ecosystem modelling applications.  The Biome-BGC ESI workflows is used to study and assess the complex relationships among ecosystem services in landscape management and environmental policies as well as for ecosystem research.  A biogeochemical ecosystem model can help us to quantify a broad range of ecosystem service indicators. These indicators can be used to evaluate real ecosystems or hypothetical reference ecosystem simulations, and can be applied for comparison of different climate or land use scenarios. These newly developed measures include: annual wood increment, yearly production of grasslands or croplands, total average carbon stock, annual evapotranspiration, damping of ecosystem daily water outflow, living and dead biomass protecting the soil against erosion, litter and coarse woody debris decomposition rate, and humification rate in the soil. They are unrealistic or even impossible to measure in the field so they have to be calculated. |
| Who is it for? | Scientists and stakeholders interested in ecology, especially in uptake of the concept of ecosystem services and ecosystem service indicators. |
| How does it work? | The calculation of the ecosystem service indicators is based on indicator specific algorithms and aggregation functions of internal model variables of Biome-BGC. The Biome-BGC Project Database & Management System (BBGCDB) supports users to manage and share all input and parameter files required for execution of Biome-BGC simulations in a workflow framework. |
| Expected results | Structured time-series (daily, monthly and/or annual) output text files, zipped and attached to the BBGCDB project record. BBGCDB project ID, URL and standard annual summary output are outputs of the workflow. |
| Links to workflows and user’s documentation | purl: <http://purl.ox.ac.uk/researchobj/myexp-687> (whole family pack)  myExperiment: <http://www.myexperiment.org/workflows/3682>  Portal: <https://portal.biovel.eu/workflows/289>  Documentation: <https://wiki.biovel.eu/x/JoOk> |
| Example of use | To create timelines of provisioning and regulating Ecosystem Service indicators (ESI) of productivity (GRaSS/WOOD – biomass materials), annual net primary production (ANPP – global climate regulation), litter and course woody debris decomposition rate (DECOMP – soil formation and composition), damping of ecosystem on daily water outflow (DESDWO – hydrological cycle and water flow maintenance) from 1901 to 2000 at Hegyhátsál meteorology field station in West-Hungary. Based on Biome-BGC simulations of various ecosystems (arable land, dry grassland, oak forest and scots pine forest), under the same site conditions with the same meteorology input. |
| Partners and *contributors | [Centre for Ecological Research](http://www.okologia.mta.hu/en/node/2), Academy of Sciences (MTA ÖK), Hungary  [University of Eastern Finland](http://www.uef.fi/)  *[Finnish Environment Institute (SYKE)](http://www.syke.fi/en-US), Finland  *EC FP7 [OpenNESS project](http://www.openness-project.eu/) |

[Back to top](#T2expTOP)

| Workflow | **Biome-BGC Sensitivity Analysis workflows (SA)** |
| --- | --- |
| Family | Ecosystem Modelling |
| What is it for? | A meteorology driven biogeochemical ecosystem model, Biome-BGC simulates the carbon, water and nitrogen fluxes and pools of terrestrial ecosystems. The Biome-BGCMuSo variant performs more realistic simulations in terms of soil hydrology, and improved ecosystem management options. Performance, success or failure of these models are highly dependent on parameter settings and variation. Due to the high number of parameters and the non-linear behaviour of the models, there are limited options to find the ‘best’ parametrization. Sensitivity Analysis (SA) is one of the ways to enhance deeper understanding for better parametrization of the model according to the model-data-fusion approach.  This workflow assists to: check the effect of parameter variability on the results; find the most influential parameters on the resulted outputs; restrict the number of parameters to decrease the degree of freedom of the model simulation; understand model behaviour. |
| Who is it for? | Scientists interested in Biome-BGC and professional users of the model for calibrated simulation. |
| How does it work? | SA requires a prior execution of a Biome-BGC Monte Carlo Experiment (MCE) that performs an independent parameter variation within given parameter ranges. Parameters, range of parameter values, output variables and number of randomized repetitions has to be set in the Biome-BGC MCE workflow. This runs off-line because of the time consuming nature of MCE jobs; potentially, several days. Then one or several SA can be launched based on the results of Biome-BGC MCE completed before. |
| Expected results | Table of sensitivity values of selected Biome-BGC output variables depending on investigated parameters and bar charts. |
| Links to workflows and user’s documentation | purl: <http://purl.ox.ac.uk/researchobj/myexp-687> (whole family pack)  myExperiment: <http://www.myexperiment.org/workflows/4490.html>  Portal: <https://portal.biovel.eu/workflows/300>  Documentation: <https://wiki.biovel.eu/x/igEtAQ>  For Biome-BGC Monte Carlo Experiment workflow, see:  myExperiment: <http://www.myexperiment.org/workflows/3620.html>  Portal: <https://portal.biovel.eu/workflows/48>  Documentation: <https://wiki.biovel.eu/x/GQA8AQ>  For Biome-BGC Model-Data-Fusion framework, see: <https://wiki.biovel.eu/x/bAEtAQ> |
| Example of use | Twenty parameters of two model versions were analysed and compared in a Biome-BGC Monte Carlo Experiment and SA. The sensitivity of gross primary production (GPP) of both models were high for specific leaf area (SLA), leaf N in RUBISCO, C:N ratio of leaves and fixation rate of N parameters. However, sensitivity of GPP’s differ considerably. Biome-BGCMuSo is much less sensitive to SLA, while the opposite can be observed in the case of some other ecophysiological parameters. |
| Partners and *contributors | [Centre for Ecological Research](http://www.okologia.mta.hu/en/node/2), Academy of Sciences (MTA ÖK), Hungary  *[Croatian Forest Research Institute](http://www.sumins.hr/en/), Croatia  *[Department of Meteorology](http://nimbus.elte.hu/index-en.html), Eötvös Loránd University, Hungary  *[Laboratory of Parallel and Distributed Systems (MTA SZTAKI)](https://www.lpds.sztaki.hu/), Hungary  *[Szent István University](http://sziu.hu/), Hungary |

[Back to top](#T2expTOP)

| Workflow | **Biome-BGC Generalized Likelihood Uncertainty Estimation workflow (GLUE)** |
| --- | --- |
| Family | Ecosystem Modelling |
| What is it for? | A meteorology driven biogeochemical ecosystem model, Biome-BGC simulates the carbon, water and nitrogen fluxes and pools of terrestrial ecosystems. It works with lots of ‘a priori’ unknown and hard to obtain model parameters. Therefore the parameterization is a critical step of using the model. Parameters can be estimated using inverse calibration techniques based on measurement data, which means that the model is being calibrated. Measurement data have to be collected with respect to the model in order to compare them. Comparison is based on misfit measure (e.g. a sort of likelihood value) which is the function of the difference between observed and modelled data. It is based on Bayesian calibration with Monte Carlo search. Each parameter is varied randomly within its ‘a priori’ range and the model is run several times using variable model parameters. Then the ‘a priori’ distribution is updated with model information (distribution of the likelihood function) to define ‘a posteriori’ density function. From the maximum of the ‘a posteriori’ density function optimal parameter values can be calculated that have the best fit to observations. |
| Who is it for? | Scientists interested in Biome-BGC and professional users of the model for calibrated simulation. |
| How does it work? | GLUE requires a prior execution of a Biome-BGC Monte Carlo Experiment (MCE) that performs an independent parameter variation within ‘a priori’ parameter ranges. Parameters, range of parameter values, output variables and number of randomized repetitions has to be set in the Biome-BGC MCE workflow. This runs off-line because of the time consuming nature of MCE jobs; potentially, several days. Then one or several GLUE analysis can be launched based on the results of Biome-BGC MCE completed before. |
| Expected results | Parameter setting and calculated likelihood values for each simulation run instances. |
| Links to workflows and user’s documentation | purl: <http://purl.ox.ac.uk/researchobj/myexp-687> (whole family pack)  myExperiment: <http://www.myexperiment.org/workflows/4481.html>  Portal: <https://portal.biovel.eu/workflows/507>  Documentation: <https://wiki.biovel.eu/x/BgA8AQ>  For Biome-BGC Monte Carlo Experiment workflow, see:  myExperiment: <http://www.myexperiment.org/workflows/3620.html>  Portal: <https://portal.biovel.eu/workflows/48>  Documentation: <https://wiki.biovel.eu/x/GQA8AQ>  For Biome-BGC Model-Data-Fusion framework, see: <https://wiki.biovel.eu/x/bAEtAQ> |
| Example of use | To produce “dotty plots” of GLUE analysis for multiple parameters of Biome-BGCMuSo model, representing ‘a posteriori’ characteristics of these parameters. Allows to identify, for example narrower (more certain) ‘a posteriori’ parameter mean and range than ‘a priori’ one at the canopy light extinction coefficient parameter. |
| Partners and *contributors | [Centre for Ecological Research](http://www.okologia.mta.hu/en/node/2), Academy of Sciences (MTA ÖK), Hungary  *[Croatian Forest Research Institute](http://www.sumins.hr/en/), Croatia  *[Department of Meteorology](http://nimbus.elte.hu/index-en.html), Eötvös Loránd University, Hungary  *[Laboratory of Parallel and Distributed Systems (MTA SZTAKI)](https://www.lpds.sztaki.hu/), Hungary  *[Szent István University](http://sziu.hu/), Hungary |

[Back to top](#T2expTOP)

| Workflow | **Microbial Metagenomic Trait Calculation and Statistical Analysis Workflow (MMT)** |
| --- | --- |
| Family | Metagenomics |
| What is it for? | The MMT workflow calculates a number of ecologically interesting traits of bacterial communities as observed by high throughput metagenomic DNA sequencing. Metagenomic traits workflows are significant as it is very likely that environmental sequencing in natural and disturbed habitats will become a routine part of monitoring programs.  This workflow allows standardized, repeatable, and comparable utilization of metagenomic data while hiding the complexity of analytical procedures.  Furthermore, inter-trait relationships can be used as habitat descriptors or indicators of artefacts during sample processing. Overall, these metagenomics community traits approaches, here combined in a single workflow helps to interpret metagenomics data to gain a full understanding of microbial community patterns in a rigorous ecological framework. |
| Who is it for? | Users who have microbial metagenomic DNA sequence data. |
| How does it work? | The workflow computes the multivariate statistics related to the metagenomic community traits outlined in Barberan et al. (2012) Exploration of community traits as ecological markers in microbial metagenomes. doi: [10.1111/j.1365-294X.2011.05383.x](http://dx.doi.org10.1111/j.1365-294X.2011.05383.x). The traits included in the statistical analyses range from GC-content to functional diversity, and deliver a set of ecological markers to discriminate between habitats or geographic locations. |
| Expected results | Data for each trait and summarizing statistics. |
| Links to workflows and user’s documentation | purl: <http://purl.ox.ac.uk/workflow/myexp-4489.3>  myExperiment: [http://www.myexperiment.org/work- flows/4489.html](http://www.myexperiment.org/work-%20flows/4489.html) (for the full calculation and statistical analysis of all traits of a new quality controlled metagenome)  Portal: Not presently loaded  myExperiment: <http://www.myexperiment.org/workflows/3349.html> (For the statistical analysis of pre-calculated metagenomic traits)  Portal: <https://portal.biovel.eu/workflows/703>  Documentation: <https://wiki.biovel.eu/x/04mD> |
| Example of use | The workflow has been used to build the Metagenomic Trait Database (<http://mb3is.megx.net/mg-traits>).  Analysis of sequenced water samples from Ocean Sampling Day, a world- wide coordinated microbial sequencing initiative. |
| Partners and *contributors | Max Planck Society, [MPI for Marine Microbiology](http://www.mpi-bremen.de/en/), Germany  *EC FP7 [Micro B3 project](https://microb3.eu/) |

[Back to top](#T2expTOP)

| Workflow | **Bioinformatic analysis of Metagenomic ampliconS workflow (BioMaS)** |
| --- | --- |
| Family | Metagenomics |
| What is it for? | BioMaS is a bioinformatics pipeline supporting biomolecular researchers to carry out taxonomic profiling studies of environmental microbial communities through target-oriented metagenomic surveys. It includes all the computational processing steps from raw Next Generation Sequencing (NGS) data input and cleaning to the final taxonomic identification and representation. In its current version, BioMaS allows the analysis of both bacterial and fungal environments starting directly from the output of 454 and illumina NGS platforms. |
| Who is it for? | Users who have microbial target-oriented metagenomic DNA sequence data generated by NGS platforms. |
| How does it work? | BioMaS includes the following consecutively and automatically running modules: i) raw data statistical and quality evaluation and arrangement for the next analysis steps; ii) mapping of sequence data on reference databases and parsing of resulting hits; iii) taxonomical binning and assignment. |
| Expected results | BioMaS produces a graphical tree and several pie-charts describing the taxonomic complexity of the investigated microbiota at different ranks (from phylum to species). All the analysis results are embedded in a single html file. |
| Links to workflows and user’s documentation | url: <https://www.biodiversitycatalogue.org/services/71>  myExperiment: <http://www.myexperiment.org/workflows/4538.html>  Portal: not applicable  Documentation: <https://wiki.biovel.eu/x/GwABAQ>  <http://testjst.ba.infn.it/openacces/BiOMaS_hELP/Biomas-PostProcessingTools.zip> |
| Publications | Fosso et al. (2015) BioMaS: A modular pipeline for Bioinformatic analysis of Metagenomic ampliconS. BMC Bioinformatics 16:203. doi: [10.1186/s12859-015-0595-z](http://dx.doi.org/10.1186/s12859-015-0595-z) |
| Example of use | Meta-barcoding high-throughput-sequencing work. Ocean Sampling Day, a worldwide coordinated microbial sequencing initiative of ocean habitats. |
| Partners and *contributors | [Institute of Biomembranes and Bioenergetics (IBBE)](http://www.ibbe.cnr.it/), National Research Council (CNR), Italy  [National Institute of Nuclear Physics (INFN)](http://www.ba.infn.it/), Italy |

[Back to top](#T2expTOP)

| Workflow | **Phylogenetic workflows** |
| --- | --- |
| Family | Phylogenetics |
| What is it for? | Phylogenies can be used as a basic tool to summarize biodiversity, to categorize groups of organisms and to study the impact of environmental change on biodiversity. The phylogenetics family of workflow and services allows performing phylogenetic inferences for systematics research.  Bayesian phylogenetic inference workflows are for performing phylogenetic inference for systematics and diversity research. Bayesian methods guide selection of the evolutionary model and a post hoc validation of the inference is also made.  Phylogenetic partitioning of the diversity across samples allows study of mutual information between phylogeny and environmental variables. This is done with the PDP workflow, using both categorical and phylogenetic information.  MSA-PAD workflow performs a multiple DNA sequence alignment coding for multiple/single protein domains invoking two alignment modes: Gene and Genome.  SUPERSMART services provide an integrated solution for the phylogenetic inference of large time calibrated trees. Public data resources are mined for suitable molecular sequence data, which is subsequently processed to form a starting point for phylogenetic inference using Bayesian and Maximum-Likelihood methods. Inferred trees can then be time-calibrated using fossil data.  Furthermore, services for the enrichment and conversion of data resources in standard data formats for phylogenetic analysis are available (NeXML parser and coder). These services can be integrated into phylogenetic inference workflows. |
| Who is it for? | Scientists interested in performing phylogenetic inference and to perform phylogenetic diversity analysis. Students who seek for a low- threshold solution to perform phylogenetic inference tasks. |
| How does it work? | Phylogenetic inference is performed using MrBayes software and checking MCMC convergence of the tree parameter (powered by GeoKS) and evaluating post hoc the fit of the model with a posterior predictive test.  Three variants of the bayesian phylogenetic workflow exist, that differ on the mode to define model of substitution:   - Automated partitioned model definition using PartitionFinder - Guide the user in the choice of partitioned model using a graphic user interface - User specified Nexus file with included full model descriptions   The phylogenetic partitioning of the diversity across samples is done using the phylogenetic entropy proposed by Chao, Chiu and Jost (2010) and equating beta diversity to mutual information between species and environment vectors.  The SUPERSMART services provide functionality to infer phylogenies from a given list of taxa of interest. A set of DNA sequences for phylogenetic inference is then assembled by querying the GenBank database. To tackle the computational challenges of inferring trees with hundreds or thousands of taxa, phylogenetic inference is accomplished in a multi-step procedure using the Maximum-Likelihood estimator implemented in ExaML and the multi-species multi-locus coalescent approach implemented in *BEAST. Computations are performed in parallel. |
| Expected results | Phylogenetic inference produces:   - Phylogenetic Tree diagrams,species-level chronograms - Posterior probability distribution of the evolutionary modelled - Posterior predictive probability of a good fit of the model - Sets of aligned DNA marker sequences for given taxa of interest   Phylogenetic diversity partitioning produces:   - HTML report including table of gamma, alpha and beta phylogenetic diversity and entropy, and graphical overlook of the contribution of each branch to the overall phylogenetic beta entropy - XML representation of the table - PhyloXML or NeXML representation of the tree and the branch beta contribution.   Data enrichment and extraction services produce documents encoded in standard (phylogenetic) file formats: NeXML, NEXUS, FaSTa, PhYLiP, Newick, Stockholm. |
| Links to workflows and user’s documentation | Bayesian phylogenetic inference workflows  purl: <http://purl.ox.ac.uk/researchobj/myexp-370>  myExperiment: <http://www.myexperiment.org/workflows/3411.html>  <http://www.myexperiment.org/workflows/3408.html>  <http://www.myexperiment.org/workflows/3410.html>  <http://www.myexperiment.org/workflows/3407.html>  Portal: <https://portal.biovel.eu/workflows/466>  <https://portal.biovel.eu/workflows/549>  <https://portal.biovel.eu/workflows/550>  <https://portal.biovel.eu/workflows/525>  Documentation: <https://wiki.biovel.eu/x/R4Ok>  PDP workflow  purl: <http://purl.ox.ac.uk/workflow/myexp-3570.5>  myExperiment: <http://www.myexperiment.org/workflows/3569.html> <http://www.myexperiment.org/workflows/3570.html>  Portal: <https://portal.biovel.eu/workflows/434>  <https://portal.biovel.eu/workflows/71>  MSAPAD workflow, Gene mode  purl: <http://purl.ox.ac.uk/workflow/myexp-4549.1>  myExperiment: <http://www.myexperiment.org/workflows/4549.html>  Portal: <https://portal.biovel.eu/workflows/712> (access on request)  MSAPAD workflow, Genome mode  purl: <http://purl.ox.ac.uk/workflow/myexp-4551.1>  myExperiment: <http://www.myexperiment.org/workflows/4551.html>  Portal: <https://portal.biovel.eu/workflows/713> (access on request)  NeXML parser and coder: <https://www.biodiversitycatalogue.org/services/70>  SUPERSMART service: <https://www.biodiversitycatalogue.org/services/78> |
| Publications | Sandionigi A, Vicario S, Prosdocimi EM, Galimberti A, Ferri E, Bruno A, Balech B, Mezzasalma V, Casiraghi M: Towards a better understanding of Apis mellifera and Varroa destructor microbiomes: Introducing “PhyloH” as a novel phylogenetic diversity analysis tool. Mol Ecol Resour 2014 doi: [10.1111/1755-0998.12341](http://dx.doi.org/10.1111/1755-0998.12341).  Vos R, Biserkov J, Balech B, Beard N et al. Enriched biodiversity data as a resource and service. Biodiversity Data Journal 2: e1125 (16 Jun 2014) doi: [10.3897/BDJ.2.e1125](http://dx.doi.org/10.3897/BDJ.2.e1125) |
| Example of use | In collaboration with ZooplantLab, Università di Milano “Bicocca”, BioVeL partners looked at the relationship of gut microbiomes in 22 pairs of host-parasites (*Apis mellifera*, honey bees and *Varroa destructor*, a parasitic mite) across seven beehives in Northern Italy. Strong similarities were found within each pair and within beehives, noting that few lineages of bacteria remain unique to either bees’ guts or mite guts. |
| Partners and *contributors | [National Institute of Nuclear Physics (INFN)](http://www.ba.infn.it/), Italy  [Institute for Biomedical Technologies (ITB)](http://www.itb.cnr.it/) and [Institute of Biomembrane and Bioenergetics (IBBE)](http://www.ibbe.cnr.it/), National Research Council (CNR), Italy  [Naturalis Biodiversity Center](http://www.naturalis.nl/en/), The Netherlands  *[The Antonelli Lab](http://www.antonelli-lab.net/), University of Gothenburg, Sweden |

[Back to top](#T2expTOP)
