# Supplementary material for: BioVeL: a virtual laboratory for data analysis and modelling in biodiversity science and ecology
Source: BMC Ecol. 2016 Oct 20;16:49. doi: 10.1186/s12898-016-0103-y (PMC5073428; doi:10.1186/s12898-016-0103-y)
Supplement: Supplementary file 4 — Additional file 4. Scientific studies using the BioVeL platform. [file 12898_2016_103_MOESM4_ESM.docx]

**Supplementary Information**

**Scientific studies using the BioVeL platform**

**1. Studying effects of climate change and other factors on biodiversity with ecological niche models:** We designed and used a workflow for ecological niche modelling (‘Generic ENM workflow’) to study the effect of environmental change or anthropogenic pressure on biodiversity and ecosystems, with particular reference to potential for non-indigenous species invasion and changes in native species distributions. Our Generic ENM workflow was supplemented with the data refinement workflow (DRW) [1] to help us carry out taxonomic name resolution, occurrence data retrieval and data preparation tasks prior to niche modelling. A post-analytical statistical workflow (ESW DIFF) allowed us to quantify the extent and intensity of change in species’ potential distributions by computing the differences between two model projections on the same geographical region but with different environmental scenarios. A typical example of using these workflows in combination is illustrated in Supplementary Figure 1.


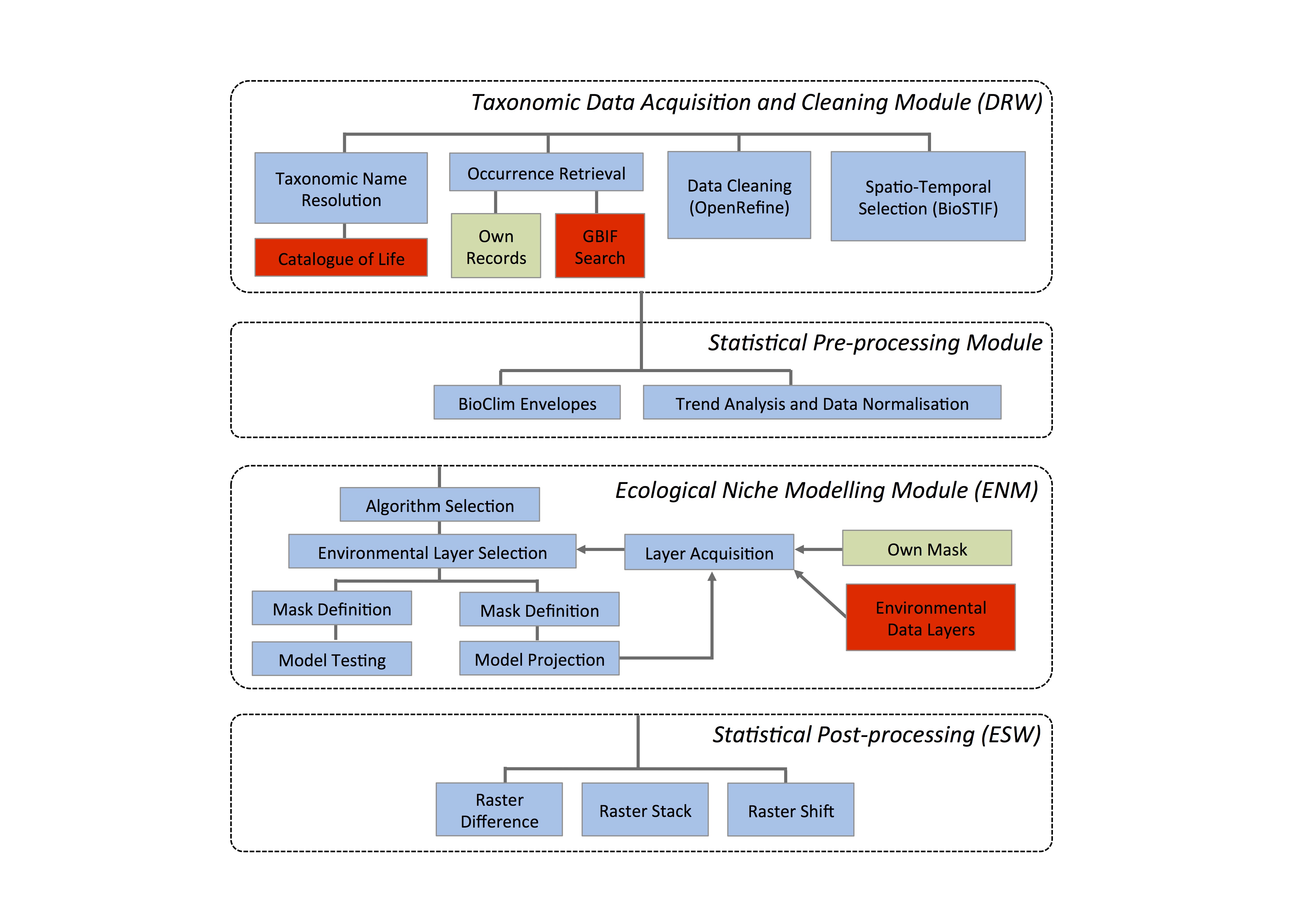


Supplementary Figure 1: Example of how several workflows can be used in combination. The diagram illustrates the principal functionalities that support ecological niche investigations using statistical correlations. The capabilities combined in the workflows support exploration of public data sources, integration with personal data, and automated analysis. Dotted lines enclose the functionalities within a workflow, while boxes describe Web service and other functions. Green boxes are input files. Red boxes are data services. Blue boxes are analytical services.

*Marine ecosystems*

We studied the distribution patterns under recent and future climate change scenarios (2050) for a guild of marine isopod crustaceans (*Idotea spp*.) and the effect of these on their host seaweeds *Fucus vesiculosus* and *F. radicans*, as well as their fish predator, the three-spined stickleback (*Gasterosteus aculeatus*) [2]. We showed that suitable habitats for these isopods seem to be more determined by temperature than by salinity patterns. Future predictions for *Idotea spp*. showed a northern shift into the Bothnian Bay, potentially increasing the extinction risk for the endemic *F. radicans*, which is more susceptible to grazing pressure than its mother species *F. vesiculosus*. Both *Fucus spp*. and *Idotea spp*. are genetically less variable in the Baltic Sea, which decreases their capacity to deal with rapid environmental changes (such as increased sea surface temperature, decreased salinity, and eutrophication) as is predicted over the next 40 years by a regional climate model. We suggested that the evolutionary potential of *Fucus* and *Idotea* populations may be the key factor in determining how they will cope with future environmental changes.

We used four workflows, including our Generic ENM workflow to analyse the potential spread of 18 marine non-indigenous species, having their origin in the East Atlantic or Pacific Oceans along a European shipping route by unregulated discharge of ships’ ballast water [3]. We showed that contrary to current risk assessment methods, temperature and sea ice concentration determine habitat suitability for 61% of species, rather than salinity (11%). We showed high habitat suitability (hot spot) for invasive species in the Skagerrak and the Kattegat, a transitional zone for invasive species entering or leaving the brackish Baltic Sea.

*Forest ecosystems*

We also used our Generic ENM workflow to analyse changes in the distribution of major European forest insect pests under different climate change scenarios. Extreme weather patterns such as storms and drought generate conditions that decrease the vitality of trees or increase the number of spots with damaged or freshly felled trees as focal points for insect attacks. Warmer winters and extended summers promote reproduction and vitality of insects because temperature is the most important environmental factor influencing insect behaviour, distribution, development, survival and reproduction [4]. Insect outbreaks and shifts in distribution alter structure and stability of forest ecosystems.

Forest insect, insect outbreak and host tree observations came from GBIF and the European Forest Institute (EFI) - Alterra Database on Forest Disturbances in Europe [5]. Host tree distribution was used as one of the environmental parameters predicting insect distribution. In a 2-step dynamic analysis we modelled historical (until year 2000) and future distribution of pest insects (*Ips typographus*, *Bupalus piniarius*, *Dendrolimus pini*, *Erannis defoliaria*, *Lymantria monacha*, *Operophtera brumata*, *Panolis flammea*, *Tortrix viridana*) by first predicting the expected changes in host tree distribution (*Picea abies*, *Pinus sylvestris*, *Betula pendula*, *Quercus robur*, *Prunus padus*). We have been able to show (Supplementary Figure 2, “currentLayer”) that, in general across all species the most suitable habitats have been in central Europe until now. Habitat suitability for (and hence potential for forest damage to be caused by) most major forest insect pests in Europe is likely to spread about 600-800 km towards the north east by the year 2050 (Supplementary Figure 2, “predictionLayer” and “diffLayer”).


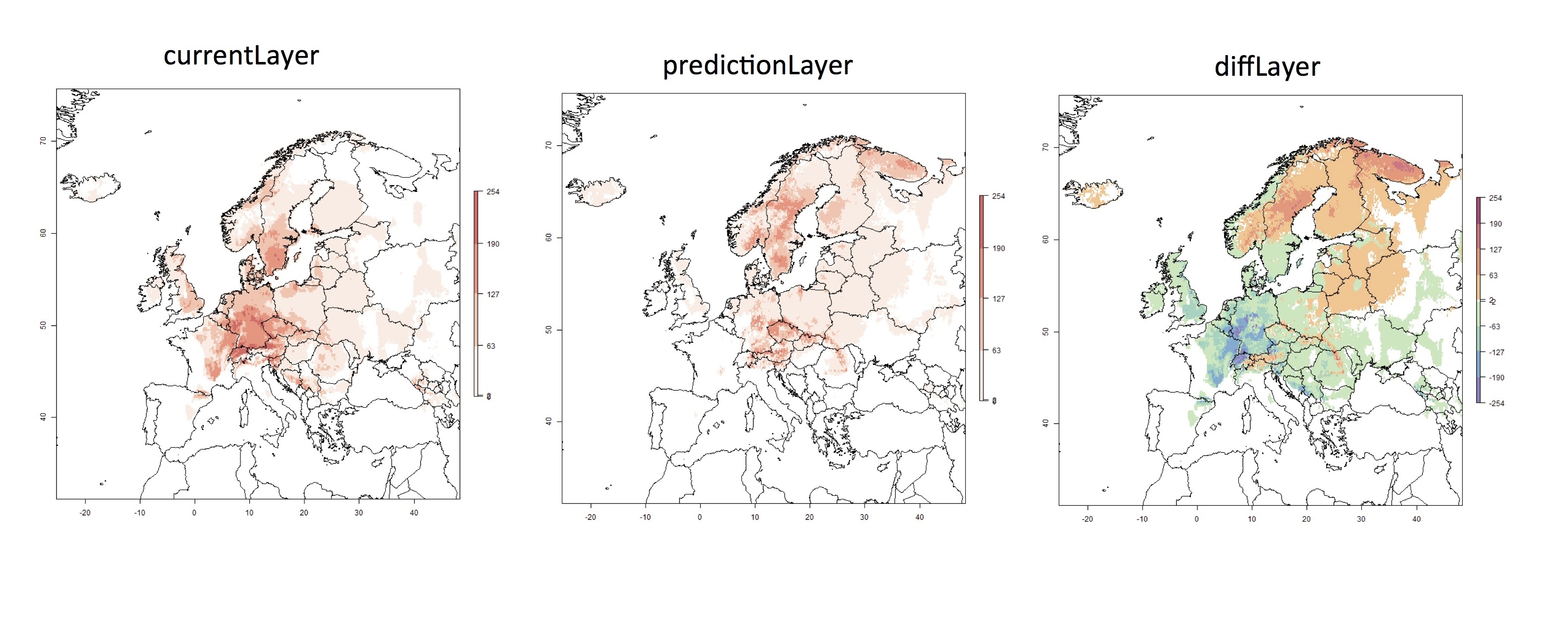


Supplementary Figure 2: Predicted shift in distribution of the European spruce bark beetle, *Ips typographus*, based on modelled distribution of its host tree, the Norway spruce, *Picea abies*. The top map (currentLayer) illustrates the historical distribution in 2000 and the middle map (predictionLayer) shows the projected distribution in 2050. In both maps, deeper red indicates a greater density of occurrence. The bottom map (diffLayer) depicts the extent and magnitude of change in distribution between 2000 and 2050. In this map colours from green to red indicate an increase and from green to blue a decrease in the predicted potential occurrence of the species. The projected potential distribution shifts from main present-day central European areas approximately 600-800km towards the north east.

The projected distribution shift is affecting Scandinavia and NE Russia, with greatest significance for Finland. Such projections are useful, for example for ecosystem management, forest protection, and forest health management planning. However, the picture is not quite so straightforward when individual species are considered, with shifts in balance between candidate species. Moreover, natural barriers, land use, forest management and human activity in general may affect the actual distribution of forest insects. Further, more detailed studies are still needed and these can be complemented by *in-silico* experimentation on prospective range shifts.

**2. Studying carbon cycling and ecosystem service indicators with biogeochemical models:** We designed several workflows for ecosystem modelling, with the aim to support simulation of plant growth and carbon / water / nitrogen balance of different natural and managed ecosystems (forests, grasslands, croplands). We re-used existing computational software variants of the Biome-BGC model: Biome-BGC v4.1.1 and the newly developed Biome-BGCMuSo [6]; wrapping these as workflows and adding capabilities to automatically perform wide-ranging data and parameter variation (sweeping). Biome-BGC is a popular, state-of-the art, mechanistic biogeochemical model that can be used to estimate different, temporally changing state variables (pools) of a specific ecosystem, different fluxes (transport) within the soil-plant system, and also fluxes between the ecosystem and the atmosphere. The improved Biome-BGCMuSo model is able to simulate interactions between the ecosystems and the climate, the changing chemical composition of the atmosphere, the different land-use management practices and disturbances, and also the changes of nutrient supply. We selected 22 output variables as a kind of ’carbon standard dataset’ i.e., Net Primary Production, Total Ecosystem Respiration, Net Ecosystem Exchange, Total Vegetation Carbon and so on to be output by the workflows. These workflows, supported by a Biome-BGC Projects Database and Management System [7] are illustrated in Supplementary Figure 3.


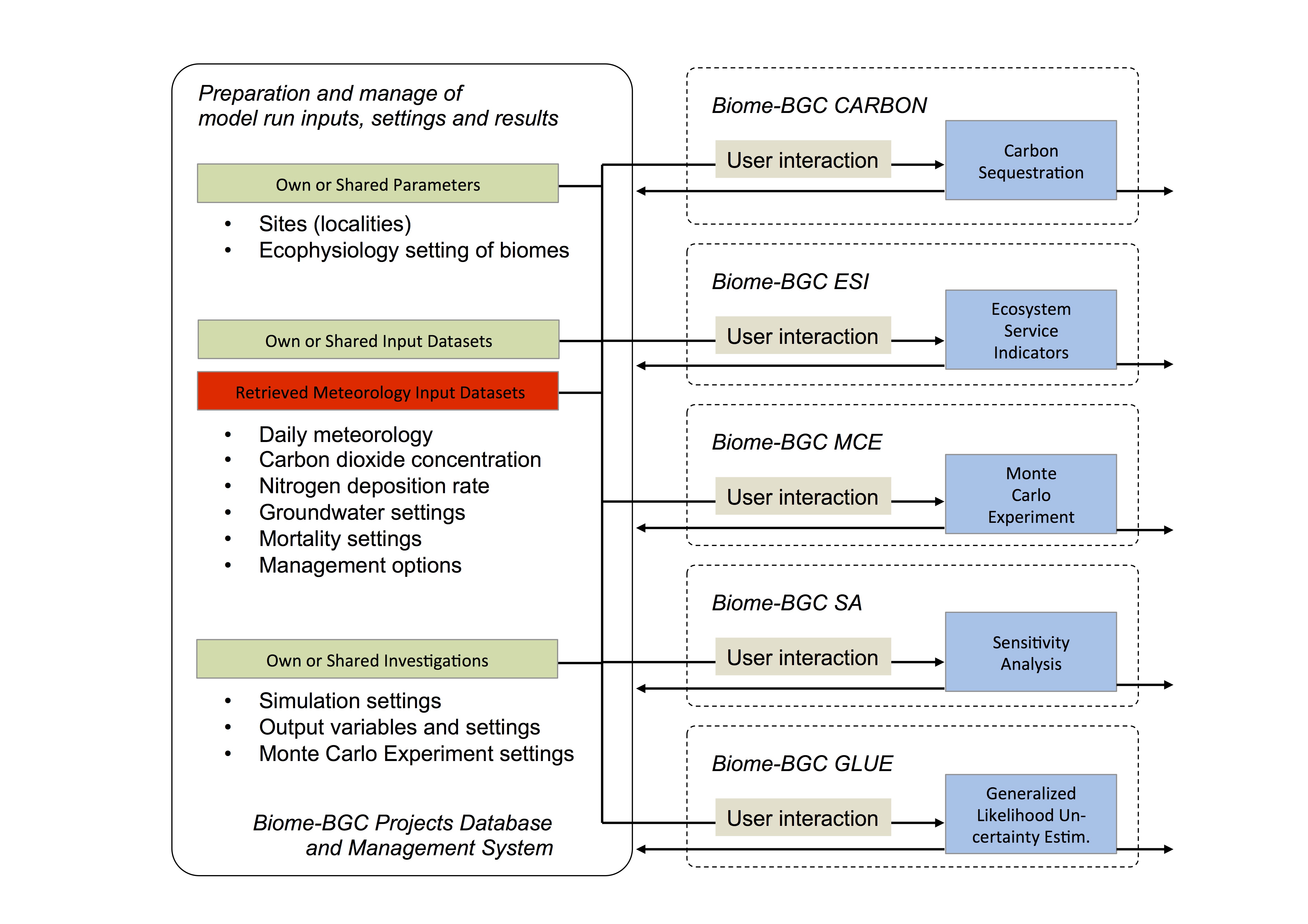


Supplementary Figure 3: Workflows for ecosystem modelling. The diagram illustrates the principal workflows and the Biome-BGC Projects Database and Management System they interact with. Dotted lines enclose the functionalities within a workflow, while boxes describe Web service and other functions. Green boxes are input files. Red boxes are data services. Blue boxes are analytical services.

As part of the MACSUR (Modelling European Agriculture with Climate Change for Food Security) project of the European Joint Programming Initiative for Agriculture, Climate Change, and Food Security (FACCE-JPI), Biome-BGCMuSo was used for a comparison of grassland models. This study assessed the capability of current models to simulate grassland functioning and their possible response to climate change [8, 9]. One of 9 models used within the comparison, Biome-BGCMuSo was used to simulate carbon (C) and water fluxes for five semi-natural grasslands sites in Europe [10]. These sites present a comparable range of climatic and agricultural management conditions. We helped ecosystem researchers to calibrate Biome-BGCMuSo against measured eddy-covariance flux data at the experimental sites. Model performance was assessed using uncalibrated (blind) and calibrated simulations. After calibration, model comparison with measurement data gave satisfactory results. For weekly-aggregated gross primary production and ecosystem respiration R^2^ was ~0.6-0.9, for daily evapotranspiration R^2^ was ~0.5-0.85 and for soil water content in the topsoil R^2^ was ~0.1-0.6. For Biome-BGCMuSo the bias was limited to the range -11 to 8 g C m^-2^ week^-1^ for C fluxes and -0.2 to 0.8 mm d^-1^ for water fluxes. The sensitivity of simulated fluxes and state variables to changes in atmospheric carbon dioxide concentration ([CO_2_]), temperature and precipitation indicate that C fluxes are dominated by [CO_2_] and temperature gradients, and are less due to plant-water relationships. Results of the study indicate that further improvement is needed in the representation of soil processes that strongly influence the biogeochemical cycles of managed and unmanaged grasslands.

The Biome-BGC ESI workflow estimates a set of biophysical ecosystem service indicators (ESIs) for a given ecosystem of a geographical location or area. Most of the provisioning, regulating and cultural ecosystem services are unrealistic or even impossible to measure in the field, therefore proxies are generally proposed and chosen as surrogate indices [11, 12]. Internal variables of biogeochemical ecosystem models open the door for constructing such a quantitative ecosystem service measures. Indicators being developed are: annual wood production; yearly maximum of total above ground biomass of grasslands as provisioning ESI, and annual net primary production; total average carbon stock (including soil carbon) as global climate regulation ESI; energy absorption by evapotranspiration as micro and regional scale climate regulation ESI; damping of ecosystem daily water outflow as hydrological cycle and water flow maintenance regulating ESI; sum of living and dead biomass protecting the soil against erosion as mass stabilization and control of erosion regulating ESI; litter and coarse woody debris decomposition rate as soil formation regulating ESI. Thus far, this workflow is being used to create and compare timelines of provisioning and regulating indicators for arable land, grassland, oak forest and scots pine from 1901 to 2000 at Hegyhátsál greenhouse gas monitoring station in West-Hungary.

**3. Studying host-parasite relationships with phylogenetic methods:** Molecular phylogenetics can be used to infer relationships between groups of organisms using genetic data, and to study their evolution. It is a basic step to summarize biodiversity and changes due to environmental influences. We designed a family of related phylogenetics workflows covering: i) multi-domain alignment procedure for coding strands against profiles in the protein domain database, PFAM [13]; ii) selection, using PartitionFinder [14] of the appropriate evolutionary model to be used for phylogenetic inference; iii) phylogenetic inference with MrBayes [15] or RaXML [16] including test for good model fit by posterior predictive check and visualization of the resulting tree; and iv) PhyloH estimation of phylogenetic diversity partitioning (alpha, beta diversity and phylogenetic entropy) [17] and correlation with trait and sampling sites. These workflows are illustrated in Supplementary Figure 4.


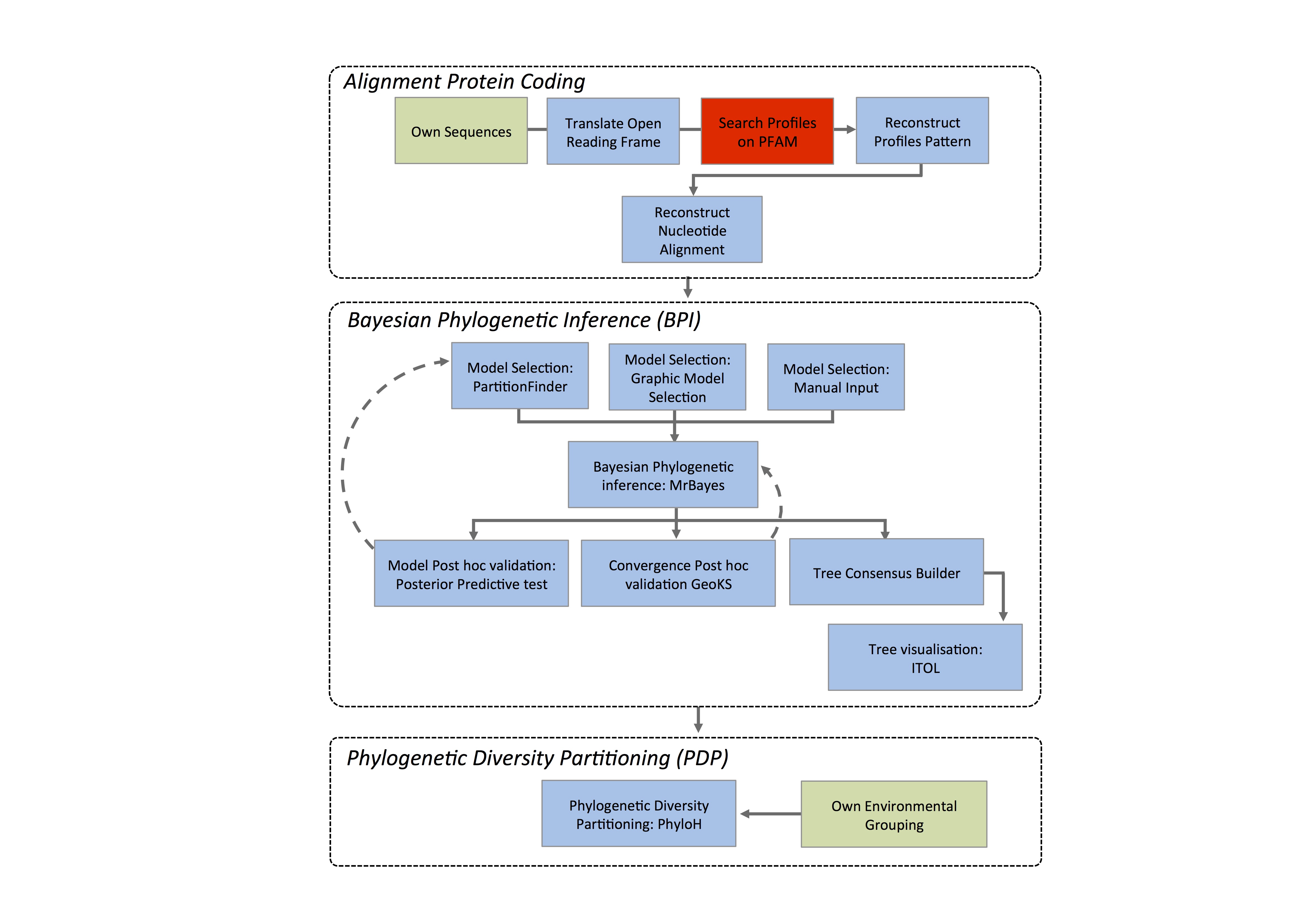


Supplementary Figure 4: Example workflows using phylogenies as a basic tool to summarize biodiversity. The diagram illustrates the principal functionalities of multi-domain alignment procedure, selection of the evolutionary model and phylogenetic inferencing, and estimation of the diversity partitioning. Dotted lines enclose the functionalities within each workflow, while boxes describe Web service and other functions. Green boxes are input files. Red boxes are data services. Blue boxes are analytical services.

By collaborating with authors of the SUPERSMART framework [18] we have been able to make available several services to format data and to mine the primary PhyLoTA database [19] in order to build a species phylogeny.

Using these workflows we looked at the relationship of gut microbiomes in 22 host-parasite pairs (*Apis mellifera* and *Varroa destructor*) across 7 beehives in Northern Italy [17]. *Varroa* is a parasitic mite of honeybees, having significant economic impact on industries that are reliant on bees. Our results showed that healthy honeybees have a simplified microbiome, constituted of few bacterial OTUs (Operational Taxonomic Units) while *Varroa* is characterized by a more complex microbiome, qualitatively not different from that of parasitized honeybees. The entropy method used, in conjunction with phylogenetic constraint as implemented in PhyloH, was able to discriminate *Varroa* microbiome from that of the parasitized honeybees (Supplementary Figure 5).





Supplementary Figure 5: Turnover of the bacterial microbiome between *Apis mellifera* and *Varroa destructor*, estimated as mutual information between read counts and microbiome environment. The external stacked histogram cyan and red shows the relative number of sequence counts assigned to different strains of bacteria found in *A. mellifera* and *V. destructor* respectively. The inferred tree that connects the strains has a branch coloured cyan to indicate significant turnover of descendant tips. The background sector of each branch is coloured from yellow to red proportional to their contribution to total turnover

There is a dramatic change of the honeybee microbiome when *Varroa* occurs, with strong similarities within bee-mite pairs. Bacteria occurring more rarely act as a clear signature to discriminate between beehives. A simple conclusion is that this parasite influences its host microbiome, with *Varroa* microbes infecting honeybees. However, the most abundant OTUs of parasitized honeybees do not derive directly from the mite, but are generalist or environmental bacteria. This is peculiar, suggesting that the strains found belonging to *Anoxybacillus* and *Lactobacillus* genera may play the role of a pioneer species, with a potential pathogenic activity.

The first two workflows (Multiple Sequence Alignment based on PFAM Accessed Domain Information (MSA-PAD) and Bayesian Phylogenetic Inference (BPI)) of Supplementary Figure 4 have been re-used to automate phylogenetic inference in a study of phylogeography, and primary/secondary plant host interaction in the pathogenic bacteria *Phytoplasma solani* (Mollicutes) [20].

**References**

1. Mathew C, Güntsch A, Obst M, Vicario S, Haines R, Williams AR, et al. **A semi-automated workflow for biodiversity data retrieval, cleaning, and quality control.** *Biodivers data J* 2014;**2**:e4221 doi:10.3897/BDJ.2.e4221.

2. Leidenberger S, De Giovanni R, Kulawik R, Williams AR, Bourlat SJ. **Mapping present and future potential distribution patterns for a meso-grazer guild in the Baltic Sea.** *J Biogeogr* 2015;**42**:241–254 doi:10.1111/jbi.12395.

3. Leidenberger S, Obst M, Kulawik R, Stelzer K, Heyer K, Hardisty A, et al. **Evaluating the potential of ecological niche modelling as a component in marine non-indigenous species risk assessments.** *Mar Pollut Bull* 2015;**97**:470–87 doi:10.1016/j.marpolbul.2015.04.033.

4. Bale JS, Hayward SAL. **Insect overwintering in a changing climate**. *J Exp Biol* 2010;**213**:980–994 doi:10.1242/jeb.037911.

5. Schelhaas M, Schuck A, Varis S, Zudin S. *Database on Forest Disturbances in Europe (DFDE): Technical Description*. European Forest Institute; 2003: http://www.efi.int/files/attachments/publications/ir_14.pdf. Accessed April 14, 2015.

6. Hidy D, Barcza Z, Haszpra L, Churkina G, Pintér K, Nagy Z. **Development of the Biome-BGC model for simulation of managed herbaceous ecosystems**. *Ecol Modell* 2012;**226**:99–119 doi:10.1016/j.ecolmodel.2011.11.008.

7. **Biome-BGC Projects Database and Management System**. http://ecos.okologia.mta.hu/bbgcdb. Accessed March 21, 2016.

8. Sándor R, Ma S, Acutis M, Barcza Z, Ben Touhami H, Doro L, et al. **Uncertainty in simulating biomass yield and carbon-water fluxes from grasslands under climate change**. *Adv Anim Biosci* 2015;**6**:49–51.

9. Sándor R, Barcza Z, Hidy D, Lellei-Kovács E, Ma S, Bellocchi G. **Modelling of grassland fluxes in Europe: Evaluation of two biogeochemical models**. *Agric Ecosyst Environ* 2016;**215**:1–19 doi:10.1016/j.agee.2015.09.001.

10. Sándor R, Acutis M, Barcza Z, Doro L, Hidy D, Köchy M, et al. **Multi-model simulation of soil temperature, soil water content and biomass in Euro-Mediterranean grasslands: uncertainties and ensemble performance.** *Eur J Agron* 2016 doi:10.1016/j.eja.2016.06.006

11. Boyd J, Banzhaf S. **What are ecosystem services? The need for standardized environmental accounting units**. *Ecol Econ* 2007;**63**:616–626 doi:10.1016/j.ecolecon.2007.01.002.

12. Kandziora M, Burkhard B, Müller F. **Interactions of ecosystem properties, ecosystem integrity and ecosystem service indicators—A theoretical matrix exercise**. *Ecol Indic* 2013;**28**:54–78 doi:10.1016/j.ecolind.2012.09.006.

13. Balech B, Vicario S, Donvito G, Monaco A, Notarangelo P, Pesole G. **MSA-PAD: DNA Multiple Sequence Alignment Framework based on PFAM Accessed Domain Information.** *Bioinformatics* 2015;**31**:2571–3 doi:10.1093/bioinformatics/btv141.

14. Lanfear R, Calcott B, Ho SYW, Guindon S. **Partitionfinder: combined selection of partitioning schemes and substitution models for phylogenetic analyses.** *Mol Biol Evol* 2012;**29**:1695–1701 doi:10.1093/molbev/mss020.

15. Ronquist F, Teslenko M, van der Mark P, Ayres DL, Darling A, Höhna S, et al. **MrBayes 3.2: efficient Bayesian phylogenetic inference and model choice across a large model space.** *Syst Biol* 2012;**61**:539–42 doi:10.1093/sysbio/sys029.

16. Stamatakis A. **RAxML-VI-HPC: maximum likelihood-based phylogenetic analyses with thousands of taxa and mixed models.** *Bioinformatics* 2006;**22**:2688–2690 doi:10.1093/bioinformatics/btl36.

17. Sandionigi A, Vicario S, Prosdocimi EM, Galimberti A, Ferri E, Bruno A, et al. **Towards a better understanding of Apis mellifera and Varroa destructor microbiomes: introducing “phyloh” as a novel phylogenetic diversity analysis tool.** *Mol Ecol Resour* 2014;**15**:697–710 doi:10.1111/1755-0998.12341.

18. Antonelli A, Condamine FL, Hettling H, Nilsson K, Nilsson RH, Oxelman B, et al. **SUPERSMART: ecology and evolution in the era of big data**. *PeerJ Prepr (update Cit before publn)* 2014;**2**:e501v1 doi:10.7287/peerj.preprints.501v1.

19. Sanderson M, Boss D. **The PhyLoTA Browser: processing GenBank for molecular phylogenetics research**. *Syst Biol* 2008;**57**:335–346 doi:10.1080/10635150802158688.

20. Delić D, Balech B, Radulović M, Lolić B, Karačić A, Vukosavljević V, et al. **Vmp1 and stamp genes variability of “Candidatus phytoplasma solani” in Bosnian and Herzegovinian grapevine**. *Eur J Plant Pathol* 2015 doi:10.1007/s10658-015-0828-z.
